# Why? What? How? Using an Intervention Mapping approach to develop a personalised intervention to improve adherence to photoprotection in patients with Xeroderma Pigmentosum

**DOI:** 10.1080/21642850.2020.1819287

**Published:** 2020-10-27

**Authors:** Jessica Walburn, Kirby Sainsbury, Lesley Foster, John Weinman, Myfanwy Morgan, Sam Norton, Martha Canfield, Paul Chadwick, Bob Sarkany, Vera Araújo-Soares

**Affiliations:** a School of Cancer & Pharmaceutical Sciences, King's College London, London, UK; b Population Health Sciences Institute, Faculty of Medical Sciences, Newcastle University, Newcastle upon Tyne, UK; c National Xeroderma Pigmentosum Service, Guy’s and St. Thomas’ Hospital NHS Foundation Trust, London, UK; d Health Psychology Section, Institute of Psychiatry, Psychology and Neuroscience, King's College London, London, UK; e Centre for Behaviour Change, University College London, London, UK

**Keywords:** Photoprotection, adherence, Intervention Mapping, behaviour change, Xeroderma Pigmentosum

## Abstract

**Background:** Intervention Mapping (IM) is a systematic approach for developing theory-based interventions across a variety of contexts and settings. This paper describes the development of a complex intervention designed to reduce the dose of ultraviolet radiation (UVR) reaching the face of adults with Xeroderma Pigmentosum (XP), by improving photoprotection. XP is a genetic condition that without extreme UVR photoprotection, leads to high risk of developing skin cancer.

**Methods**: The IM protocol of 6 steps was applied, involving comprehensive mixed-methods formative research. Key stakeholders (XP clinical staff and Patient and Public Involvement Panel), were instrumental at every step. Behaviour change methods were informed by the IM taxonomy, therapeutic approaches (e.g. ACT, CBT) and coded according to the taxonomy of behaviour change techniques (version 1).

**Results:** We designed a personalised modular intervention to target psychosocial determinants of photoprotective activities that influence the amount of UVR reaching the face. Content was developed to target determinants of motivation to protect and factors preventing the enactment of behaviours. Participants received personalised content addressing determinants/barriers most relevant to them, as well as core ‘behaviour-change’ material, considered important for all (e.g. SMART goals). Core and personalised content was delivered via 7 one-to-one sessions with a trained facilitator using a manual and purpose designed materials: Magazine; text messages; sunscreen application video; goal-setting tools (e.g. UVR dial and face protection guide); activity sheets. Novel features included use of ACT-based values to enhance intrinsic motivation, targeting of emotional barriers to photoprotection, addressing appearance concerns and facilitating habit formation.

**Conclusion**: IM was an effective approach for complex intervention design. The structure (e.g. use of matrices) tethered the intervention tightly to theory and evidence-based approaches. The significant amount of time required needs to be considered and may hinder translation of IM into clinical and non-academic settings.

Poor adherence to treatment for dermatological conditions is widespread (Ahn, Culp, Huang, Davis, & Feldman, 2017). Despite photoprotection being the only way to avoid damage from ultraviolet radiation (UVR), which leads to skin cancers, adherence rates are poor in at-risk populations (Nahar et al., 2016; von Schuckmann et al., 2019). Interventions designed to improve photoprotection have had only moderate success (Geller et al., 2018). The use of theory in intervention design has been patchy, with a lack of detail on how theory was applied, and few examples of change in theoretical constructs being measured (Taber et al., 2018; Wu et al., 2016). This critique would apply to many interventions across health behaviour research and is the backdrop to consistent calls for improvements in design and reporting standards (Craig et al., 2008; Hoffmann et al., 2014; O’Cathain et al., 2019).

This paper describes the development of a theory-based intervention to improve adherence in a condition requiring maximum photoprotection. Xeroderma Pigmentosum (XP) is an extremely rare genetic condition caused by faulty nucleotide excision repair, whereby UVR damage cannot be corrected (Fassihi et al., 2016). Consequently, people with XP are at considerable risk of skin and eye cancers (Bradford et al., 2011; Brooks et al., 2013). There is no cure and the only way to prevent cancers is to constantly rigorously photoprotect from UVR in daylight. Optimal adherence means lowering overall exposure (i.e. staying indoors as much as realistically possible) and meticulously protecting when outdoors. This involves wearing clothing to shield the face (i.e. a legionnaire-style face visor with a transparent UVR protective screen or a combination of wide-brimmed hat, glasses, scarf or face buff) and the body (i.e. long sleeves and trousers or skirt), alongside regular application of broad spectrum factor 50 sunscreen. Consistently adhering to this regime is extremely challenging. Patients find it uncomfortable, hot, time-consuming and restrictive in terms of family, work and social functioning (Anderson, Walburn, & Morgan, 2017; Morgan, Anderson, Walburn, Weinman, & Sarkany, 2019). Most adults do not achieve the required photoprotection levels (Sainsbury, Vieira et al., 2018; Walburn, Canfield et al, 2019).

We aimed to develop an intervention to improve photoprotection in adults with XP, which would be suitable for future delivery by healthcare professionals during routine clinical care. Due to the extreme nature of photoprotection required and the existence of no prior psychosocial research, we chose not to rely on a single theory or restrict the intervention to determinants observed in other at-risk populations, such as melanoma survivors. We used Intervention Mapping (IM; Bartholomew Eldredge, Markham, Kok, Ruiter, & Parcel, 2016), which is a systematic framework designed to integrate theory and evidence into interventions and has been used to target adherence across multiple conditions (e.g. Heath, Cooke, & Cameron, 2015; Zwikker et al., 2012). To date, excluding one intervention to change behaviour in a preschool setting (Tripp, Herrmann, Parcel, Chamberlain, & Gritz, 2000), IM has not been applied to photoprotection activities. This paper gives a transparent description of the intervention development process using IM and conforms to TIDieR guidelines (see supplementary file 1; Hoffmann et al., 2014).

## Methods

This section briefly summarises the six steps proposed by IM, and how these were applied to photoprotection. Please refer to the IM manual for a more detailed explanation of the steps (Bartholomew Eldredge et al., 2016).

### Step 1: Logic model of the problem

The logic model is a depiction of the hypothesised causal relationships that underpin the behaviour in need of change.

As this was the first adherence intervention in the XP population, we conducted a comprehensive needs assessment with a broad theoretical base. It involved a mixed-methods programme of research, which included four studies (Walburn et al., 2017) . All aimed to identify determinants of photoprotection that could be modified by a behaviour change intervention. Potential psychological determinants selected for investigation were identified from reviews of previous studies of photoprotection in other at-risk populations (Wu et al., 2016) and the general population (Bruce, Theeke, & Mallow, 2017), theories from the wider adherence literature such as the updated Common-Sense Model of self-regulation (CSM; e.g. illness perceptions, necessity beliefs and concerns about photoprotection, automaticity; Leventhal, Phillips, & Burns, 2016) and the Theoretical Domains Framework (TDF; e.g. beliefs about capabilities, beliefs about consequences, intention, emotion, social influences; Cane, O’Connor, & Michie, 2012). The TDF is not a theory; instead, it is a collection of constructs taken from a review of 33 individual theories. This approach is consistent with recommendations to use wide-ranging frameworks in intervention design due to overlapping constructs in single models (Araújo-Soares, Hankonen, Presseau, Rodrigues, & Sniehotta, 2019). The Patient and Public Involvement (PPI) panel and clinical stakeholders also contributed potential determinants (e.g. influence of family, friends and social interactions). All participants were recruited from the XP specialist service at Guy’s and St Thomas’ NHS Foundation Trust. The following briefly summarises the methods for each study (see individual publications for further details):

- A qualitative study to gain an in-depth understanding of individuals’ experiences of photoprotection and XP in daily life. Two researchers interviewed 47 people with XP (n=25 adults, n=22 children or carers). An inductive thematic framework analysis was undertaken to examine patterns within and across cases to form explanatory accounts (Morgan et al., 2019).
- An N-of-1 study to identify intra-individual variation in photoprotection and its psychological determinants within adult patients (n=22) using ecological momentary assessment (EMA). Participants completed an electronic diary each evening for 50 days, composed of single-item questions measuring psychological constructs most likely to vary over time; for example, self-regulatory (e.g. effort, experienced barriers), environmental (e.g. weather, risk perception), cognitive-emotional (e.g. negative thoughts about photoprotection, feeling self-conscious, stress, mood, mental exhaustion, energy), and motivational constructs (e.g. importance of photoprotection, self-efficacy), and social support (Sainsbury, Vieira et al., 2018).
- A cross-sectional survey of 156 patients (adults, children or carers) in Western Europe and U.S.A. to assess psychosocial determinants of photoprotection in a large representative sample of people with XP. The survey included a mixture of standardised questionnaires and items designed specifically for this study to measure the following psychological constructs: perceptions of XP, photoprotection beliefs, intention to photoprotect, self-efficacy, automaticity, perceived social support (Walburn, Canfield et al., 2019).
- Identification of psychological determinants of the amount of UVR reaching the face (‘Dose-to-Face’ study) across 21 days in the summer months. The dose of UVR reaching the face was estimated by combining UVR exposure at the wrist with facial photoprotection worn during exposure. To capture these data, participants wore an electronic UVR dosimeter and completed a daily UVR protection diary (see supplementary file 2) to record when they were outdoors and what photoprotection was used. Psychological constructs were derived from the cross-sectional survey (Walburn et al., 2017).

*Consensus conference.* To ensure that the intervention would fit within routine care for UK XP patients and had the support of the XP clinical team, we involved the clinical team and the PPI panel in a Consensus Conference. The main purpose of the conference was to decide which drivers of photoprotection identified in the research *should be* targeted by the intervention. We followed an adapted version of the consensus methodology, based on Nominal Group Theory (Bartunek & Murninghan, 1984), used to agree changes to a diabetes self-management package (DAFNE Study Group, 2002; Dennick et al., 2016). Researchers from each study submitted determinants of photoprotection as ‘evidence statements’ which indicated whether each determinant was modifiable by psychological intervention and provided a summary of the empirical evidence. Evidence statements were synthesised into ‘intervention recommendation statements’ by JWa, and sent to attendees for review before the conference. No determinant was excluded and any conflicts in evidence were highlighted for discussion at the conference. Recommendations were discussed and approved, rejected, or amended at the conference, which was attended by key stakeholders: researchers (*n* = 10), PPI panel (*n* = 3), and the XP clinical team (*n* = 5). An independent chairperson facilitated the event. This approach produced concrete recommendations for intervention design, enabled stakeholders without specialist research knowledge to participate as equal partners in discussions, and avoided cul-de-sac debates between researchers about the value of evidence from different methodologies.

### Step 2: Programme (i.e. the intervention) outcomes and objectives – logic model of change

The target behaviour is dismantled into its constituent sub-behaviours, which are mapped to their determinants and translated into intervention change objectives using matrices. A logic model of change is generated from the change objectives to guide the selection of behaviour change strategies. We identified the multiple photoprotection behavioural outcomes (i.e. the outcome of a successful change), dissected these into specific behavioural performance objectives (i.e. what sub-behaviours need to be performed to achieve the change) and generated change objectives (i.e. the desired change in the determinant required to achieve behavioural change). We referred to the intervention recommendation statements throughout this step.

### Step 3: Intervention design

In this step, the focus moved to ‘how’ to intervene so that change occurs. We held a series of workshops to complete steps 3 and 4. Theory-based methods of behaviour change were matched to the change objectives, guided by the IM taxonomy of behaviour change methods (Kok et al., 2016) and other theories and therapeutic approaches. To aid future replication of the intervention, techniques were also mapped to the 93-item taxonomy of Behaviour Change Techniques (BCTs; Michie et al., 2013). The modes of delivery and exactly ‘how’ the behaviour change methods would be translated into intervention components were defined

### Step 4: Intervention production

Materials were co-created with key stakeholders. We followed an approach used by O’Brien et al. (2016), whereby the core intervention team developed the intervention in stages, punctuated by workshops with key stakeholders. Each had a key role in the process (i.e. intervention team: to build an effective evidenced-based intervention; PPI panel: to ensure that it is acceptable to patients; clinical team: to ensure that it is clinically accurate, appropriate, and could be integrated in the routine care pathway). We used an iterative process to move from the matrices to intervention content. Stakeholders were involved at all stages, reviewing and re-reviewing drafts of patient-facing intervention content until all were satisfied.

### Step 5: Intervention implementation plan

Tasks required to transfer the intervention to its ‘real-life’ setting (i.e. NHS XP clinical service) are beyond the scope of this paper.

### Step 6: Evaluation plan

A protocol to evaluate the efficacy of the intervention and explore the processes involved was developed (Walburn, Norton et al., 2019).

## Results

### Step 1: Logic model of the problem

The following summarises the research findings that critically influenced intervention design (see separate publications for full results). Sixty-four evidence statements were synthesised into 21 draft intervention recommendations (see supplementary file 3) for review at the consensus conference, of which 19 were approved (see Table 1).

**Table 1.** Intervention recommendation statements approved by the consensus conference.

1	**Recommendations related to photoprotection behaviour**
	To gain largest reduction in UVR dose to the face, the intervention should include tools to promote better sunscreen use, greater use of protective clothing, and target lifestyle adjustments such as time of day and duration of time spent outdoorsd duration of time spent outdoors
2	To improve photoprotection, the intervention should include tools to assess the extent and nature of changes in the level of protection within an individual. Where change results in worse protection, maintenance of better protection across different contexts and situations will be targeted.
3	To improve photoprotection, the intervention should include tools to increase awareness and insight into photoprotection behaviour.
4	**Recommendations related to beliefs**
	To improve photoprotection, the intervention should include tools to elicit and challenge doubts about the necessity of photoprotection related to negative health consequences.
5	To improve photoprotection, the intervention should include tools to elicit and challenge doubts about the effectiveness of photoprotection and emphasise that the best way to protect is to combine all the different ways to protect.
6	To improve photoprotection, the intervention should include tools to target the perception of low personal control over health consequences related to XP.
7	To improve photoprotection, the intervention should include tools to elicit the extent and nature of any concerns about photoprotection practices and include tools to manage any such concerns.
8	**Recommendations related to risk perception**
	To improve photoprotection, the intervention should include tools to target perceptions of low UVR risk in relation to time, weather and season.
9	To improve photoprotection, the intervention should include tools to counteract the belief that an absence of noticeable physical symptoms (in both burners and non-burners) means photoprotection is not required. It should sever the link between symptom experience and photoprotection behaviour and encourage photoprotection regardless of symptoms.
10	**Recommendation related to acceptance**
	To improve photoprotection in patients who are resistant to the XP identity, the intervention should include tools to promote illness acceptance.
11	**Recommendations related to motivation and habit**
	To improve photoprotection, the intervention should include tools to increase and reinforce reflective motivation to photoprotect.
12	To improve photoprotection, the intervention should include tools to target low prioritisation of photoprotection and reinforce the priority in the context of competing daily priorities.
13	To improve photoprotection, the intervention should include tools to target self-efficacy for photoprotection in the presence of personally relevant barriers
14	To improve photoprotection, the intervention should include tools to establish routines and habits.
15	**Recommendations related to social context**
	To increase the likelihood that new photoprotection behaviours will be maintained, the intervention should include tools to manage any experience of receiving (or perceiving) negative reactions from others (enacted stigma).
16	To improve photoprotection, the intervention should include tools to encourage participants to appropriately and skilfully disclose about XP, when it is acknowledged by the patient to be a barrier to photoprotection. The level of disclosure will be decided by the patient.
17	To improve photoprotection, the intervention should include tools to enhance informal social support from family and friends (e.g. adjustment of daily activities, reminders to photoprotect), if lack of support is a barrier to photoprotection.
18	**Recommendations related to psychological impact of photoprotection**
	To improve photoprotection, the intervention should include tools to target general negative low mood.
19	To improve photoprotection, the intervention should include tools to elicit the extent and nature of the relationship between emotional experiences (in the moment) and photoprotection (e.g. feeling stressed, worried, mentally exhausted) and include tools to reduce/manage the negative impact of any such emotional experiences on photoprotection.

*Non-Adherence to photoprotection for the face.* All studies reported that levels of photoprotection varied, with most participants doing less than recommended. For example, N-of-1 analysis of the UVR protection diary showed that 62% of adults used ‘very poor’ or no facial photoprotective clothing for ∼20% of outdoor time and sunscreen was not frequently applied (Sainsbury, Vieira et al., 2018). This non-adherence was likely to have clinical implications and confirmed that intervention in the adult population was needed. Three intervention recommendation statements (statements 1–3) related to photoprotection activities were approved at the Consensus Conference.

**Modifiable determinants of photoprotection adherence.**

- *Qualitative Study*. The qualitative study identified three groups of adults who differed in their response to photoprotection. A minority of adults (*n* = 4) were dominated by the daily demands of photoprotection, organised their life to avoid exposure to UVR, and had high levels of adherence, which came at considerable emotional costs. A second group (*n* = 10) habitually integrated photoprotection into everyday life. Friends and family assisted with photoprotection tasks; however, their level of photoprotection was sub-optimal. A third group of adults (*n* = 11) admitted that their photoprotection was haphazard and limited. Photoprotection was either a visible reminder of an XP identity they wished to resist, or they preferred to ‘live for today'. They experienced XP-related stigma, hid their condition, and received little helpful support (see Anderson et al., 2017; Morgan et al., 2019; Walburn, Anderson & Morgan, 2020). These findings highlighted the role of habit in enactment of activities, negative emotional consequences of photoprotection including stigma and appearance concerns, prioritisation of other needs above photoprotection, and the role of social support, as reflected in recommendation statements 9, 10, 12, 14, 15-19.
- *N-of-1 study.* N-of-1 analyses provided a detailed day-by-day understanding of intra-individual variation in photoprotection. Fluctuations in behaviour were associated with time of day and weekday versus weekend (the direction of the relationship different across individuals); photoprotection was higher if the weather was rated as sunnier and the perceived risk was greater. The need for stability of photoprotection across contexts was reflected in recommendation statement 2. Self-regulatory constructs (e.g. greater effort, fewer external barriers) and other psychological factors (e.g. fewer negative thoughts about XP, less mental exhaustion, higher energy) were positively associated with photoprotection. Stress of photoprotection, general mood, and feeling self-conscious when photoprotecting showed different relationships with protection for different individuals and were thought to be bidirectional (e.g. positive mood could be associated with worse protection – not wishing to spoil good mood with photoprotection, or as a consequence of good protection – feeling positive that higher protection was achieved) (see Sainsbury, Vieira et al., 2018). These findings informed recommendation statements 8, 9, 11, 12, 13, 15, 18, 19.
- *Dose-to-face study.* The dose of UVR reaching the face was calculated as a combination of overall UVR exposure and level of photoprotection worn during exposure. Conference participants recommended that the intervention should target both behavioural pathways. Key psychosocial factors with protective effects included holding stronger beliefs in the necessity of photoprotection and carrying out photoprotective activities automatically. Psychosocial ‘risk factors’ associated with higher UVR exposure included having stronger belief in the effectiveness of photoprotection, greater wellbeing, and being more satisfied with social support. These were reflected in statements 4–6, 16, 17, 19.
- *Cross-sectional International survey.* The following psychological variables were associated with increased likelihood of better photoprotection when outdoors: greater personal control of XP, stronger beliefs in necessity and effectiveness of photoprotection, and higher intention. There was a positive association between photoprotecting by staying indoors and greater concerns about photoprotection when outdoors, stronger concerns about having XP, and greater XP-related distress. Greater automaticity and self-efficacy were correlates of both better photoprotection when outside and staying indoors. These findings informed recommendation statements 4–8, 11, 13, 14, 18, 19 (Walburn, Canfield et al., 2019).

*Consensus Conference.* Two draft recommendations were discarded; one due to content overlap and one due to ambiguous supporting evidence. Key discussions centred around the following:

- Conference attendees recognised the complexity of photoprotection and difficulty in responding to conflicting evidence from cross-sectional and dose-to-face studies. For example, stronger beliefs about the perceived effectiveness of photoprotection were associated with better adherence to photoprotection whilst outdoors (i.e. sunscreen and clothing) in the cross-sectional survey and were also linked to higher overall exposure to UVR (i.e. going outdoors for longer and/or at a time of day when UVR is higher) in the dose-to-face study. The intervention development team still wished to address doubts about effectiveness of photoprotection, whereas other stakeholders expressed concern that this might inadvertently increase UVR risk if individuals spent more time outdoors because they had ‘too much’ confidence in the effectiveness of their photoprotection, which they might not be doing correctly (e.g. missing areas when applying sunscreen). Consensus was achieved, by the intervention team stating that they would emphasise correct use of each photoprotection activity, that multiple activities were needed for optimal protection, and that it was important to consider scheduling of time/duration outdoors (referred to as ‘Smart-Scheduling’ in the intervention).
- The negative relationship between emotional wellbeing and photoprotection reported by the qualitative and dose-to-face studies resonated with the experience of the clinical team. The PPI panel and clinical teams were adamant that, although the primary aim of the intervention was to improve adherence to photoprotection, this should not be achieved at the expense of psychological health and social interactions. The intervention development team agreed that content to protect emotional wellbeing and reduce the burden of photoprotection would be incorporated. The PPI panel and clinical teams requested that the intervention should be engaging and valued by patients.

**Figure 1.**
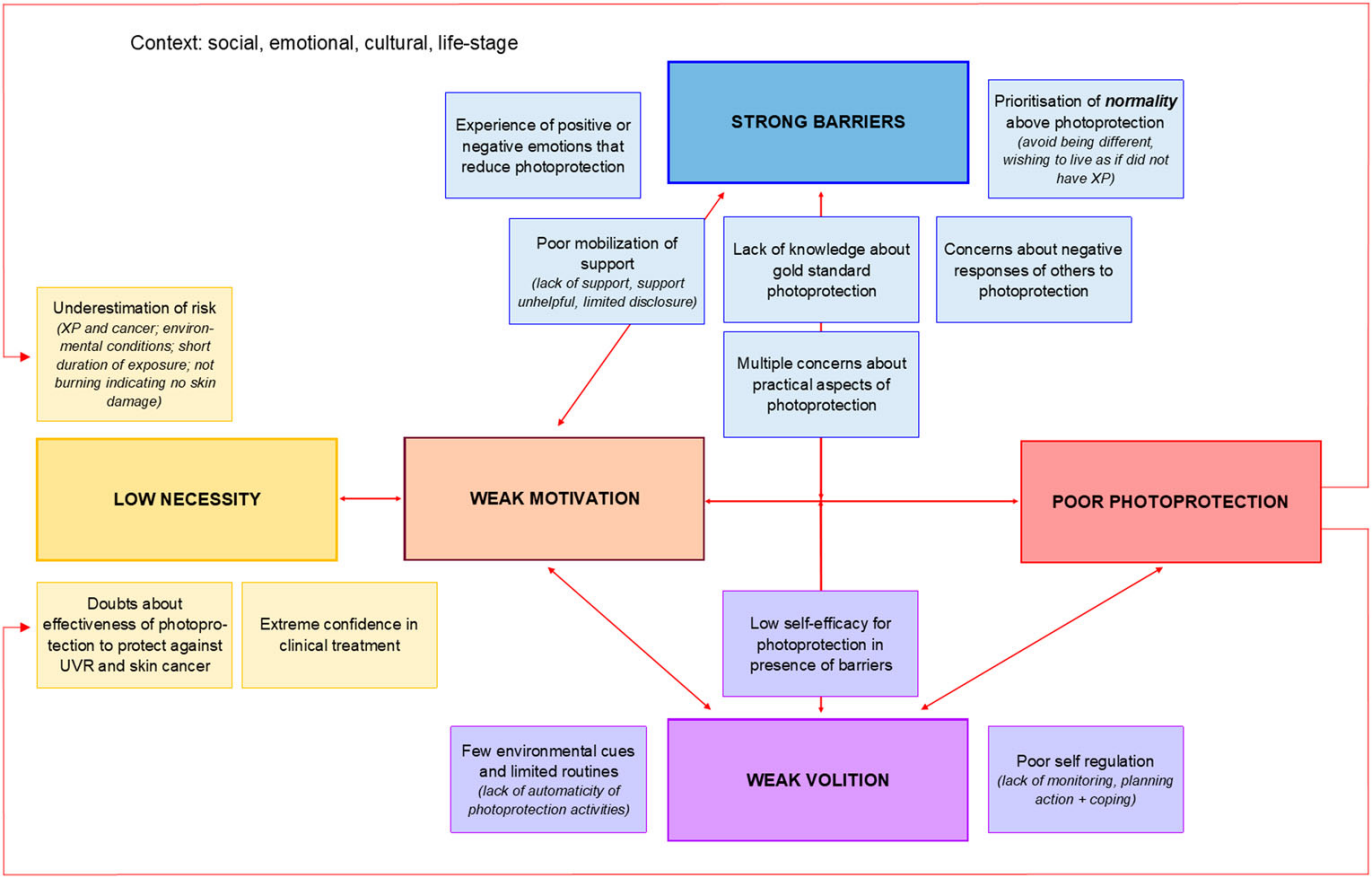
Logic model of determinants of poor photoprotection when outdoors.

*The Logic model of the problem.* We developed two logic models incorporating the recommendations from the Consensus Conference. Logic model one depicted the two behavioural pathways determining the dose of UVR reaching the face (i.e. photoprotection used outdoors and the time/duration of being outdoors; see supplementary file 4.). Adding details of determinants to both pathways was overly complex to guide intervention design, and we limited logic model two to the determinants of poor photoprotection when outdoors. The impact of intervening on all behaviours was considered and ‘photoprotection’ was used as the umbrella term for all photoprotection activities, including smart scheduling of outdoor time (Figure 1).

In this logic model, the determinants were grouped into those that influenced motivation to photoprotect, and volitional factors preventing the enactment of photoprotection, even if motivation was high. The importance of each determinant and the interrelationships would be different for each person. Beliefs contributing to doubts about the perceived necessity of photoprotection (e.g. underestimation of risk of UVR exposure related to environmental conditions such as weather) and various concerns about photoprotecting (e.g. worries about looking different) and other barriers, such as poor mobilisation of social support, were hypothesised to influence motivation to photoprotect. Volitional factors included lack of automaticity of photoprotection and poor self-regulation. Low self-efficacy for managing barriers to photoprotection influenced both motivation and volition. Motivation and volition were considered not to be mutually exclusive and the relationship between them bidirectional. The structure of the logic model was influenced by the Necessity and Concerns framework (Horne et al., 2013) and the Health Action Process Approach (Schwarzer, 2008).

### Step 2: Intervention outcomes and objectives – logic model of change

The overall objective was to reduce the dose of UVR reaching the face of adults with XP by improving photoprotection. The clinical team decided that the face should be the target of this intervention, as this is the site of the majority of skin cancers and is most difficult to protect (Kraemer, Lee, Andrews, & Lambert, 1994). According to clinical recommendations, optimal photoprotection would be wearing a face visor or a combination of wide-brimmed hat, glasses, scarf or face buff, and hoodie worn-up, plus sunscreen and lip-block, both factor 50, and minimisation of time spent outdoors. To achieve the overall objective, four behavioural objectives were defined:

1. apply the appropriate amount of broad-spectrum factor 50 sunscreen and lip-block, in the correct way, consistently before going outside.
2. re-apply sunscreen and lip-block every 2–3 hours.
3. consistently wearing an ‘excellent’ or ‘very good’ (Sainsbury, Vieira et al., 2018) combination of photoprotective clothing to protect the face.
4. adjust outside daily activities to reduce UVR exposure within realistic parameters.

For each of these behavioural objectives, we defined four performance objectives (e.g. to apply the appropriate amount of broad-spectrum sunscreen, one would need to: *Obtain broad-spectrum factor 50 sunscreen; make decision to apply the sunscreen and lip-block; apply the correct amount in the correct way; maintain sunscreen use every time you go outside*). See supplementary file 5 for the performance objectives for each behavioural objective.

**Figure 2.**
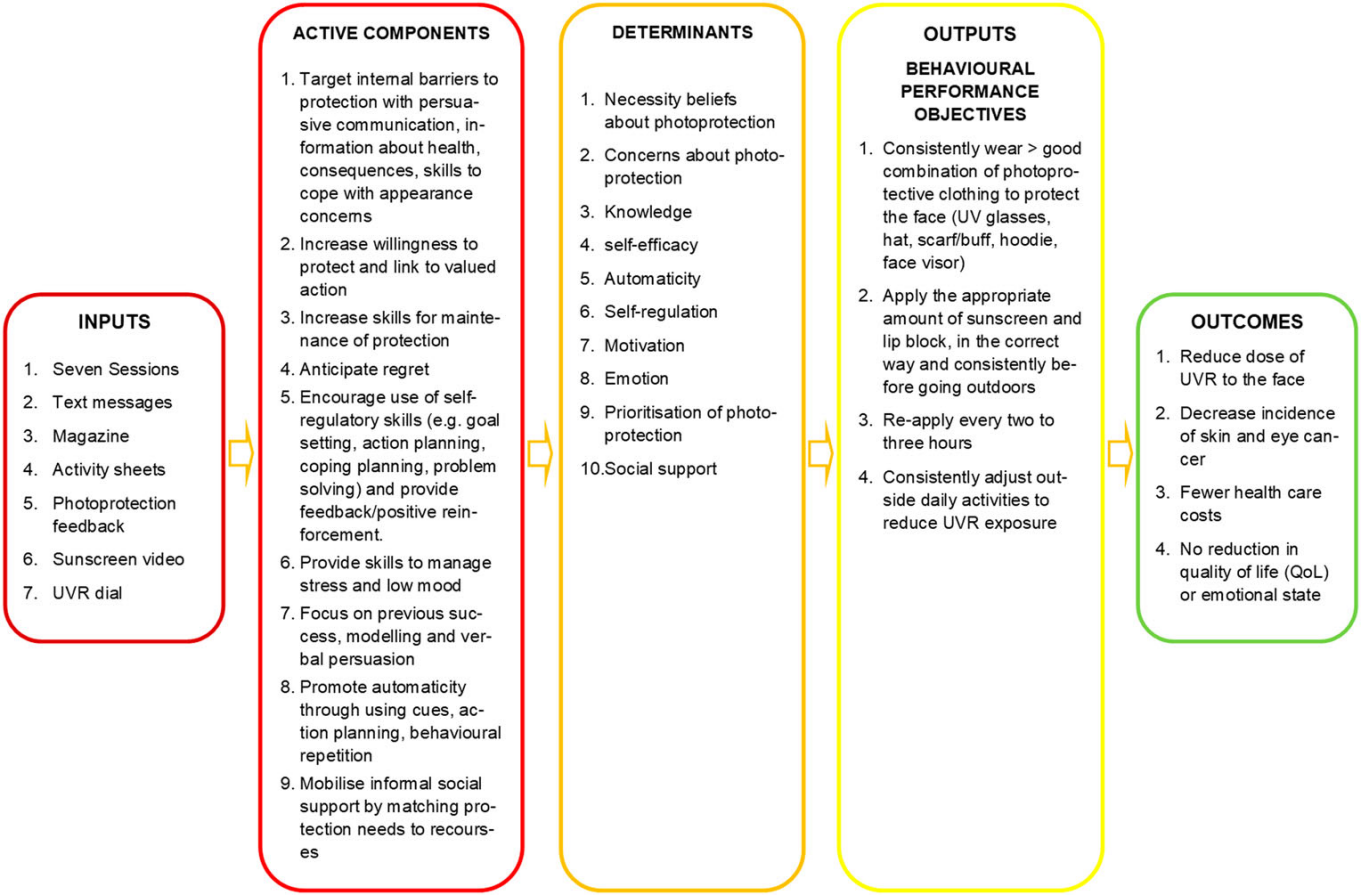
Logic model of change.

We mapped the determinants in the logic model with the performance objectives to form change objectives. For example, for the performance objective, ‘making the decision to apply sunscreen and lip-block’, we defined three change objectives for the determinant of ‘Emotion’, which covered the impact of positive and negative mood on photoprotection: *Recognise impact of emotion on decision to adjust activities and that relationship is bi-directional*; *Express confidence in ability to self-regulate own emotion to facilitate adjustment of activities; Demonstrate ability to regulate own emotion.* See supplementary file 6. for all the change objectives for determinants related to the decision to apply sunscreen/lip block performance objective. We condensed all change objectives into the logic model of change (Figure 2).

### Steps 3, 4 and 5: Design and produce the intervention; plan the intervention use including adoption, implementation, and maintenance

**Figure 3.**
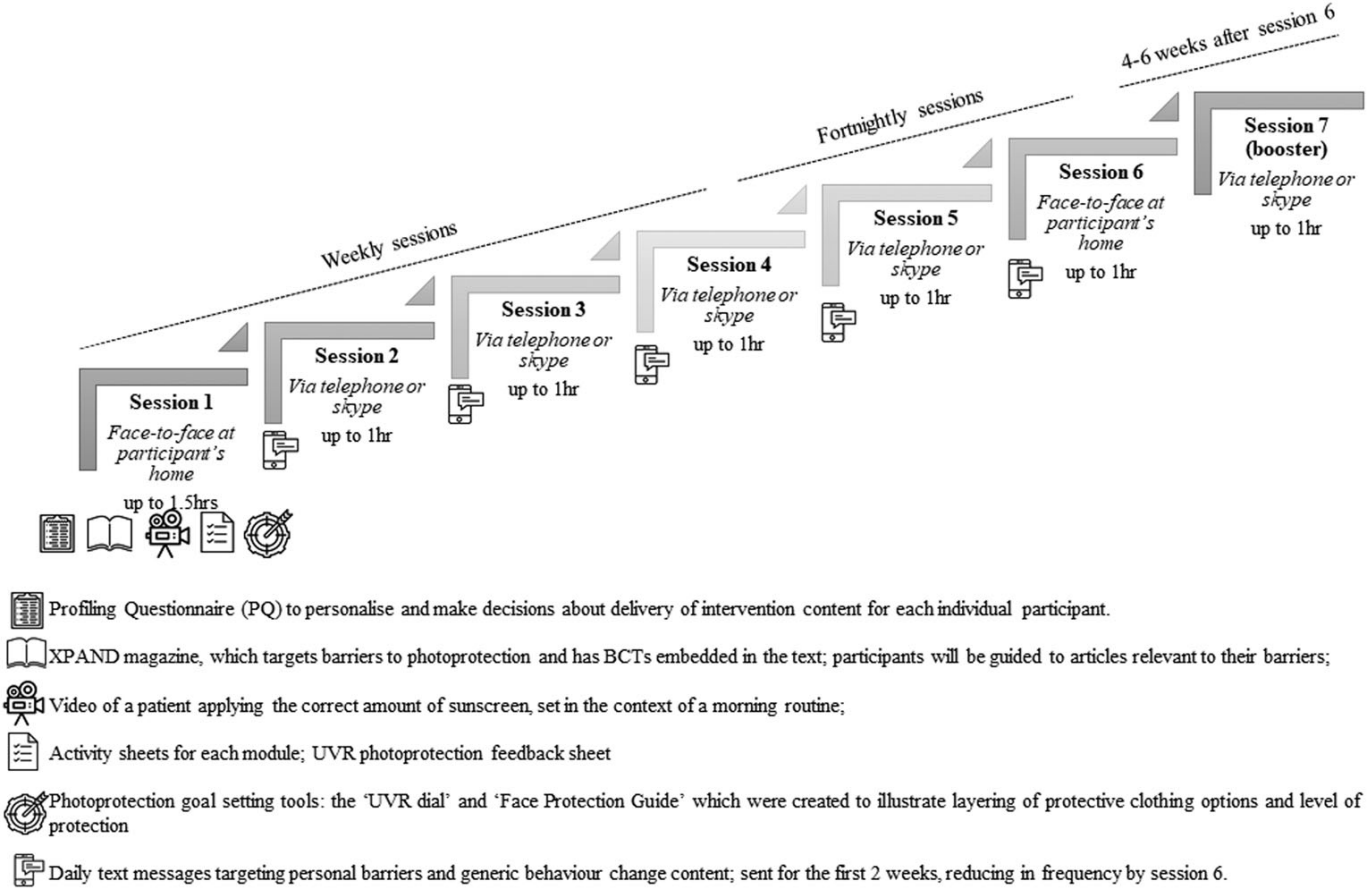
The structure of XPAND.

The intervention was called ‘XPAND – Enhancing XP Photoprotection Activities – New Directions’. It was centred around seven, one-to-one sessions across 12–14 weeks, between an intervention facilitator and participant. The content delivered was personalised to the specific photoprotection activities that the individual needed to improve (e.g. wearing a face-buff) and their pattern of motivational barriers for each activity. Core behaviour change content, which supported the enactment of photoprotection, was delivered to all participants. Personalisation was conducted with reference to data from the needs assessment (if the participant took part in phase I) and by completion of a profiling questionnaire based on the barriers detailed in the logic model, completed at baseline. Iterative tailoring continued throughout, as the facilitator adapted content to respond to changes during the intervention. For a detailed description of the process of personalisation, see our companion paper (see Sainsbury, Forthcoming). The one-to-one sessions were supported by a range of components (magazine, activity sheets, video, goal setting tools, personalised feedback on current level of photoprotection, text messages), each infused with behaviour change strategies. The structure of XPAND is shown in Figure 3.

An intervention matrix guided the design and creation of XPAND. Each change objective was mapped to relevant theories, two taxonomies of BCTs (IM taxonomy; Kok et al., 2016; BCT taxonomy v1; Michie et al., 2013), motivation and behaviour change methods, as specified in several clinical psychology approaches including Acceptance and Commitment Therapy (ACT; Hayes, Luoma, Bond, Masuda, & Lillis, 2006), and XPAND components. An excerpt from the full matrix is displayed in Tables 2 and 3. Table 2 displays core content related to development of habit to facilitate enactment of photoprotection, which was delivered across multiple components of XPAND. It included evidenced-based steps of habit formation (adapted from Gardner, Lally, & Wardle, 2012) such as the use of prompts and cues (i.e. via texts and visual prompts) and creation of implementation intentions during sessions. Habit was promoted as a strategy to ‘lighten the cognitive load’ of photoprotection as, if more automatic, it would occupy less conscious thought and resources. Table 3 shows the mapping for two change objectives related to and ‘appearance concerns’ and ‘emotion’. We anticipated that worries about appearance whilst photoprotecting would be common, because participants would be wearing potentially conspicuous clothing (e.g. face-buff) for the first time. We adapted a standard cognitive behaviour therapy protocol for people with a physical difference (Clarke, Thompson, Jenkinson, Rumsey, & Newell, 2013) to empower patients by affirming their concerns and providing practical tools to manage unwanted attention. Emotional content focussed on increasing awareness of how mood (positive and negative) and stress can interact with photoprotection and provided strategies for managing this impact, so that good protection was achieved without reduction in emotional wellbeing (as highlighted by the Consensus Conference). We leveraged the avoidance of negative emotions (e.g. worry, fear, guilt), often experienced as a consequence of not protecting (i.e. the ‘poor photoprotection hangover’), as an incentive to use better photoprotection in the future. See supplementary file 7 for an example of mapping for all core content and personalised content.

**Table 2.** Excerpt from the XPAND core content matrix, mapping a change objective for habit to theory, behaviour-change strategies and modes of delivery.


Change objective	Behaviour-change strategies mapped to taxonomies [Intervention Mapping (IM) taxonomy of behaviour change techniques (V1)]	Key theory/framework	One-to-one session	Magazine	Text messages	Video showing sunscreen application	Other materials
1. Photoprotection activities become habitual	**Intervention Mapping (IM)**: Implementation intentions;^a^ cue altering;^a^ planning coping responses^a^ (Bartholomew Eldredge et al., 2016) BCTv1: action planning; prompts/cues (7.1); habit formation (8.3) (Michie et al., 2013)	TDF (Goals) HT	Habit formation strategies: (adapted from Gardner et al., 2012; Lally & Gardner, 2013) focus on linking photoprotection to existing routines; use of prompts and cues for photoprotection that will trigger them to protect (e.g. hat by the door); explain importance of repeating the activity in the same circumstances. Facilitator helps participant make if-then statements. Facilitator will elicit participant’s own experiences of habit formation and emphasise that extra effort now will increase chances that new behaviour will become automatic and less burdensome over time.	Article including practical tips for habit formation – ‘How to make sticking to a UVR routine easier'	External prompts for new behaviour and messages were developed to reinforce concepts. ‘Putting on your sunscreen at the same time in the same place every morning will help it become habit'	Shows how to link application within existing morning routine	Goal setting record sheet: includes action and coping plans. Building blocks of behaviour change graphic shows how photoprotection activities can be developed to become habits.

**HT** Habit Theory (Verplanken, 2006; Verplanken & Aarts, 1999; Verplanken & Orbell, 2003).

**TDF** Theoretical Domains Framework (Cane et al., 2012).

Intervention Mapping evidenced-based change methods (Bartholomew Eldredge et al., 2016).

^a^Methods to change Habitual, Automatic, and Impulsive Behaviours.

^b^Basic methods at the individual level.

^c^Methods to change skills, capability, and self-efficacy and to overcome barriers.

^d^Methods to change attitudes, beliefs and outcome expectations.

^e^Methods to change awareness and risk perception.

^f^Methods to increase knowledge.

^g^Methods to change social influence.

**Table 3.** Excerpt from the XPAND personalised matrix mapping two change objectives to theory, behaviour-change strategies and modes of delivery: appearance concerns; emotion.


Change objectives	Behaviour-change strategies mapped to taxonomies [Intervention Mapping (IM) Taxonomy of behaviour change techniques (V1)]	Key theory or framework	One-to-one session (Summary of content included in the intervention manual)	Magazine	Text messages	Video showing sunscreen application	Other materials [Determinant-specific activity sheets]
1. Reduce concerns about looking different whilst photoprotecting	IM: belief selection;^b^ tailoring;^b^ modelling;^b^ planning coping responses;^b^ persuasive communication;^b^ reinforcement;^b^ self- monitoring of behaviour;^b^ reattribution training;^c^ provide opportunities for social comparison^g^ (Bartholomew Eldredge et al., 2016) BCTv1: Problem solving (1.2); instruction on how to perform the behaviour (4.1); reattribution (4.3); behavioural experiments (4.4); Information about emotional consequences (5.6); social comparison (6.2); credible source (9.1); pros and cons (9.2) (Michie et al., 2013)	TDF (beliefs about consequences; skills) NCF (concerns) CBT (attention training; social skills and coping strategies)	The aim is to affirm appearance concerns and acknowledge that they can be an important part of the daily burden of having XP. It provides practical strategies to manage unwanted attention involving diversion of attention in the moment, choosing types of protection that are more likely to blend in and boosts general social skills. Content adapted from existing manual (Clarke et al., 2013). *Manual module/s:* Concerns about appearance when photoprotecting	Article on managing barriers to photoprotection includes key strategies to manage appearance worries – ‘What’s stopping you getting the UVR protection you need?'	X	X	Activity sheet reiterates that concerns about appearance are natural; summarises tips to manage staring; gives examples relevant to photoprotection.
2. Minimise impact of positive or negative emotions that reduce photoprotection	IM: tailoring;^b^ planning coping responses;^b^ reinforcement;^b^ self- monitoring of behaviour;^c^ Improving physical and emotional states;^c^ Anticipated regret.^d^ BCTv1: Problem solving (1.2); Monitoring of emotional consequences (5.4); Anticipated regret (5.5); Information about emotional consequences (5.6); Pros and cons (9.2); Reducing negative emotion (11.2)	TDF (Skills; Emotions) CBT (stress management)	The aim is to modify emotion if it has a negative impact on photoprotection. The relationship between emotions (positive and negative) and photoprotection will be explored (i.e. not wishing to wear face-buff when feeling happy in case it lowers mood). Facilitators will provide cognitive, emotional, and behavioural strategies to manage fluctuations in mood and stress, to minimise influence on protection and improve emotional stability in the long-term. *Manual module/s:* Mood and photoprotection Stress and photoprotection	Article on managing barriers to photoprotection includes positive and negative emotions as a barrier – ‘What’s stopping you getting the UVR protection you need?'	*Feeling good today and don’t want protection to bring you down? Remind yourself how protecting now can help you to achieve the things you want in the future*.	X	Activity sheets reinforce skills and concepts discussed in the session. Content related to pleasant activity scheduling adapted from Getselfhelp.co.uk. Symptoms of low mood adapted from https://www.nhs.uk/conditions/stress-anxiety-depression/low-mood-and-depression/

**CBT** Cognitive Behavioural Therapy.

**NCF** Necessity and Concerns Framework (Horne et al., 2013).

**TDF** Theoretical Domains Framework (Cane et al., 2012).

Intervention Mapping evidenced-based change methods (Bartholomew Eldredge et al., 2016).

^a^Methods to change Habitual, Automatic, and Impulsive Behaviours.

^b^Basic methods at the individual level.

^c^Methods to change skills, capability, and self-efficacy and to overcome barriers.

^d^Methods to change attitudes, beliefs and outcome expectations.

^e^Methods to change awareness and risk perception.

^f^Methods to increase knowledge.

^g^Methods to change social influence.

*The spirit of XPAND.* Before detailing the components of the intervention, it is important to emphasise the ‘spirit’ behind XPAND. Through XPAND, we aimed to increase motivation to photoprotect and reduce concerns or barriers (internal and external) in order to ‘tip the balance’ towards better photoprotection. Motivation was boosted by encouraging participants to identify the values, informed by ACT (Hayes et al., 2006), that protecting themselves from UVR allowed them to move towards (i.e. what did photoprotection enable them to do?). These personal reasons for photoprotection were referred to throughout the sessions, when setting SMART goals and planning rewards for effort or progress. This was achieved using the ACT-informed ‘carrot and stick’ metaphor whereby the ‘sticks’ of skin damage and potential for cancer (i.e. avoidance-based) were supplemented with ‘carrots’ or future-focused, values-consistent benefits of photoprotection (e.g. being a caring and available parent or friend, being a reliable employee). As well as working with participants to better manage barriers to photoprotection, it was acknowledged that some barriers could not be totally eliminated. Therefore, XPAND emphasised the benefit of being willing to act in line with values, despite the presence of barriers. Finally, ACT ideas were also combined with the rationale for habit formation/automaticity as a way to promote emotional wellbeing by achieving balance between photoprotection and other priorities (i.e. photoprotection is the backdrop on which engagement with life happens, rather than taking time and resources away from it).

*XPAND components.* Each individual received a blend of personalised and core content, delivered via one-to-one sessions, and a suite of patient-facing materials.

*One-to-one sessions with an XPAND facilitator.* XPAND was designed to be delivered by healthcare professionals without advanced therapeutic skills in seven sessions. The ‘essence of motivational interviewing’ (i.e. collaboration, acceptance, compassion, and evocation; Miller & Rollnick, 2009) was adopted throughout. Sessions one and six were face-to-face in the individual’s home. All other sessions were conducted via Skype or phone. Sessions one-four were weekly, reducing to biweekly for five-six. Session seven took place four-to-six weeks after session six. According to the Health Behaviour Change Competency Framework (Dixon & Johnston, 2010), the content was of medium intensity and required that a clinician follow a manual and adapt it, as indicated by the needs of the patient.

**Figure 4.**
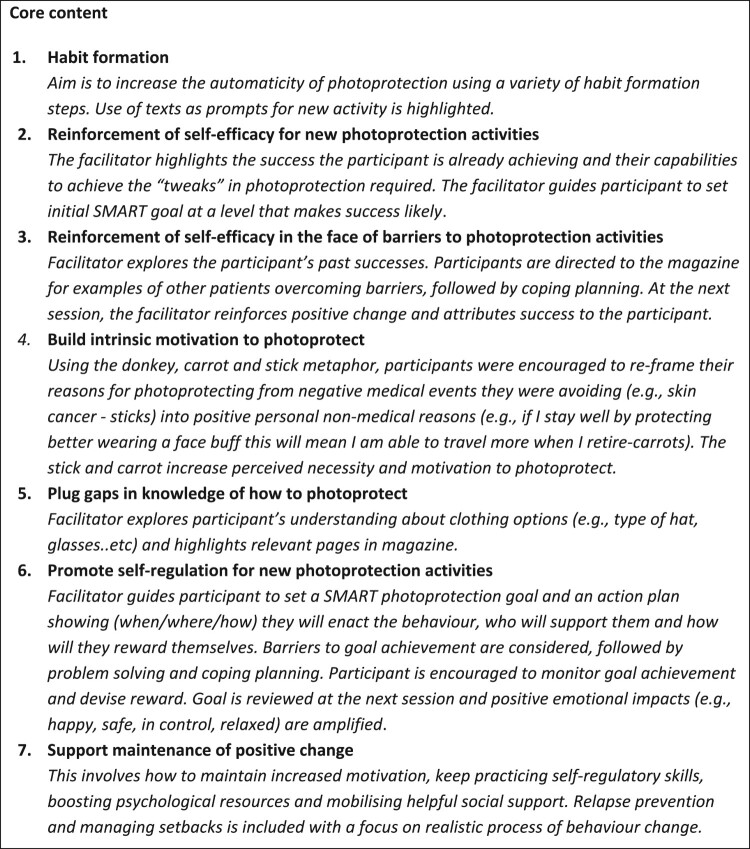
A summary of core and personalised topics.

Facilitators used the XPAND manual, which was separated into seven modules for personalised content, corresponding to the barriers in the logic model, and core (volitional) content, which included topics considered to be relevant to all (e.g. self-regulation). Each session contained a blend of core and personalised content. A summary of this content is shown in Figure 4.

*Activity Sheets.* Activity sheets with additional detail about each module or concept being discussed were developed. These could be used within the session or completed by the participant in their own time.

*XPAND Magazine.* A 44-page magazine was produced to target the determinants of photoprotection from the logic model. It was designed to be a standalone, active component of XPAND. Each article was mapped to a determinant and infused with relevant BCTS. A creative health writing team and a graphic designer worked closely with the research team to produce a high-quality, engaging, ‘consumer-format’ magazine, which included XP patient stories, information, and tips from experts. Content was reviewed by all stakeholders to ensure acceptability and accuracy. Three independent researchers checked that the final magazine included the intended BCTs, with substantial agreement (75% agreement, inter-rater reliability of 0.67 Gwet coefficient). The facilitator used the magazine within the sessions to prompt discussion and reinforce concepts discussed. Participants were guided to the articles most relevant to their own photoprotection barriers.

*Text Messages.* Text messages were sent to remind and reinforce content discussed during sessions. Individuals could opt out of the text messages and still receive the other XPAND components. Each participant received texts relating to photoprotection barriers relevant to them. Messages were selected from a bank of 76 texts, which were written for XPAND or adapted from existing interventions (Hingle et al., 2014; Janda, Youl, Marshall, Soyer, & Baade, 2013; Petrie, Perry, Broadbent, & Weinman, 2012). For example, ‘UV rays are invisible … don’t let that fool you! Protect whatever the weather, or time of day'. Consistent with evidence for habit development (Lally, van Jaarsveld, Potts, & Wardle, 2010), these were sent daily for the first two weeks, reducing in frequency until session six. Participants chose the most suitable time for messages and were encouraged to base the timing on when they would be enacting their goal-related photoprotection activity, so they could serve as a proximal reminder. The text bank is included in supplementary file 8.

*Video of sunscreen application.* A 3-minute, professionally-produced video showing either a male or female character of different ethnicities (2 versions) applying the correct amount of sunscreen demonstrated the link between application and greater effectiveness of the sunscreen (i.e. better coverage gives better photoprotection). This was highlighted by showing exposed skin left by patchy application, using a UVR camera. The video reinforced how to integrate sunscreen application into a morning routine (habit formation process) and the importance of wearing clothing alongside sunscreen to protect the face. Each participant was encouraged to watch the video in their own time. The facilitator used the video to prompt a discussion about sunscreen use and elicit any barriers to application/reapplication throughout the day.

*Photoprotection goal-setting tools.* Two tools were devised to reinforce the idea that relatively small changes in protective behaviour can result in significantly improved overall protection, and prompted discussion about how the differences in clothing, sunscreen, and the duration and timing of being outdoors can contribute to reducing the dose of UVR reaching the skin.

*UVR Dial.* This was an interactive tool consisting of moveable discs pictorially depicting each form of face photoprotection and demonstrating the layering of UVR protective measures. The outer disc was colour-coded to match the face photoprotection guide (see below). It was used by the facilitator to support the setting of SMART goals and illustrate that multiple photoprotection activities were required for optimal protection. See supplementary file 9. for a copy of the dial.

*UVR Face Protection Guide.* The ‘face protection guide’ was a risk-ruler, devised in conjunction with the clinical team to demonstrate different levels of photoprotection, from low to high, using face photoprotection clothing combinations. For example, ‘low protection’ is provided by wearing a single item: baseball cap, hoodie, face-buff/scarf, or glasses; whereas ‘high protection’ is provided by wearing a wide-brimmed hat, glasses, *and* face-buff/scarf (Sainsbury et al., 2018). It used colours to depict the adequacy of protection provided by the different combinations. Broad spectrum factor 50 sunscreen application was addressed separately. The face protection guide illustrated the inadequacy of current practices, and how protection would improve if new activities were adopted. It was used in conjunction with giving individual feedback about the dose of UVR reaching the face, recorded from the formative research. Please contact the authors to access copies of XPAND materials and the facilitator manual.

*Involvement of stakeholders in the development of XPAND.* Key stakeholders were the PPI panel (a patient, a parent and founder of the XP Support Group, and a teacher who had taught several students with XP); four adult patients with XP (2 participating in the trial and 2 not eligible as they had excellent adherence); and the clinical team from the National XP service at Guy’s and St Thomas’ NHS Foundation Trust. A summary of their involvement and impact on the creation of XPAND is shown in Table 4.

**Table 4.** Involvement of stakeholders in the development of XPAND.

Stakeholder	Component of XPAND	Key impact
PPI Panel	Structure, all patient-facing materials, and outcome measures	The number of one-to-one sessions, session spacing, and acceptability of Skype was informed by PPI panel, to balance effectiveness and participant burden. Checked acceptability of all patient-facing materials. Interviewed by health writers for inclusion in the magazine. A personal story from the patient member of the panel was featured in the magazine. The decision to limit the number of outcome measures for the trial of XPAND and to reduce the number of follow-up periods for the control group in 2019 was in response to concerns about participant fatigue.
Adults with XP	Magazine, text messages, sunscreen application video	Personal stories from each adult were included in the magazine. Acceptability of the magazine and text messages. One patient assisted the actors during filming of the video to ensure authenticity of sunscreen application.
XP Clinical team	Structure, all patient facing materials, face protection guide	Decision to use multiple modes of delivery as team advised that XPAND needs to be appropriate for heterogeneous patient group. Development of the face protection guide: the clinical team participated in a group task to rank order the combinations of photoprotection clothing Interviewed by health writers for articles, including quotes, in the magazine. Checked accuracy of the information in the magazine. Checked acceptability of text messages. One member of the clinical team was present during filming of the video to ensure depiction of sunscreen application and other clothing was consistent with recommendations from the clinical team.

*Planning for implementation.* Description of the implementation process is beyond the scope of this paper. If successful, XPAND will be incorporated into routine care by the UK XP clinical team.

### Step 6: Develop an evaluation plan

The efficacy of XPAND was tested using a randomised controlled trial (RCT) design. Adults with a diagnosis of XP, without cognitive impairment, and who displayed sub-optimal levels of photoprotection were randomised to receive the intervention in the year 2018 or to be the waitlist control group, who then received the intervention at the same time of year in 2019. The primary outcome was the dose of UVR reaching the face, assessed during two follow-up periods of 21 days in 2018 (June-to-July and August-to-September) and two follow-up periods in 2019 (March and June-to-July; delayed intervention control group only). The performance and change objectives informed the choice of outcomes and process variables (Walburn, Norton et al., 2019). Using in-depth interviews, a qualitative process evaluation assessed the acceptability of XPAND and its influence on photoprotection from the perspective of the recipient. A fidelity check of the extent to which XPAND was delivered to the manual and success at portraying the ‘spirit of XPAND’ was conducted by independent researchers. Trial results will be reported separately.

## Discussion

We were tasked with developing an effective intervention that could be delivered by existing healthcare professionals to improve adherence to photoprotection in adults with the extremely rare, genetically inherited condition, XP. The stakes were high, since this is the only way for patients to avoid skin and eye cancers and future funding for behavioural interventions is unlikely in such a rare condition. Our use of mixed-methods to conduct a comprehensive needs assessment to inform the logic model ensured that factors, important for such extreme photoprotection, were identified. This revealed that it was important to target multiple photoprotection activities, including smart-scheduling, used simultaneously by XP patients, rather than single behaviours (i.e. sunscreen application) often addressed by interventions in other at-risk populations (e.g. melanoma survivors, organ transplant recipients; Wu et al., 2016). Novel determinants identified included concerns about appearance whilst photoprotecting, emotions (positive and negative), and resistance to photoprotection in favour of other non-health priorities. Providing information about the damaging effects of UVR on appearance has been used to influence photoprotection in healthy samples (Williams, Grogan, Clark-Carter, & Buckley, 2013), whereas supporting individuals to cope with negative reactions from others was a new direction here. Adoption of a single social cognitive model would have limited XPAND to cognitive determinants of photoprotection, such as attitudes towards photoprotection (Theory of Planned Behaviour), risk perception (Health Belief Model) and self-efficacy (Social Cognitive Theory, Bandura, 2001; Wu et al., 2016), which have dominated interventions in other clinical populations. Notwithstanding that these theories have informed interventions associated with improvements in photoprotection, considerable variance remains unexplained (see Sutton, & White, 2016). By systematically facilitating habit formation across multiple modes of delivery, XPAND addresses a gap in these interventions where automatic processes have largely been ignored.

As a consequence of identifying novel determinants, we needed to select innovative approaches to modify these factors. XPAND combined theories from health psychology with methods, such as ACT (Hayes et al., 2006), more commonly used in the treatment of psychopathology. No previous photoprotection studies have used ACT (Hayes et al., 2006) in their designs. We translated core processes of values and committed action into an activity of identifying meaningful personal (non-medical) reasons for photoprotection to increase motivation and willingness, and boost necessity beliefs. We also applied ‘active acceptance’ as a way to live with the real challenges of daily photoprotection, such as the experience of negatively experienced emotions related to photoprotection. Established taxonomies (Kok et al., 2016; Michie et al., 2013) were limited when describing how XPAND targeted these factors to facilitate behaviour change. Further research needs to delineate emotional processes, so that intervention developers can select techniques and report them in standardised manner.

Systematic reviews of previous photoprotection interventions report that, where theory has been used, it is unclear *how* it has informed design, behaviour change strategies, and outcome measures (Wu et al., 2016). In contrast, the use of IM meant that XPAND’s design and content was dependent upon theory. Specifically, linking behavioural performance objectives to change objectives and translating these into intervention materials ensured that each element of the intervention was purposeful. No aspect of the design was superfluous to requirements and avoided the ‘It Seemed Like a Good Idea at The Time’ principle (Eccles, Grimshaw, Walker, Johnston, & Pitts, 2005). The IM matrix approach provided justification for each component, which was advantageous in gaining support from stakeholders and approval from research governance committees. The matrices provided a transparent audit trail for fellow designers, which should enable robust replication. Replication has been identified as a current priority for health psychology intervention research, if the discipline is to amass sufficient high-quality evidence to inform clinical practice and public policy guidelines (O'Carroll, 2014).

In common with the experiences of other researchers (Abbey et al., 2017; Heinen, Bartholomew, Wensing, van de Kerkhof, & van Achterberg, 2006; McEachan, Lawton, Jackson, Conner, & Lunt, 2008), shortage of time was the main challenge of using IM. In real-time, XPAND took a year to develop (excluding the formative research, which itself took a year to conduct and synthesise), with at least 5 months spent on developing the matrices. This was compounded by the complex nature of photoprotection, which includes multiple behaviours, each with potentially unique determinants. Due to time restraints, we combined the behaviours, unless we had evidence that the determinant was only relevant to a specific photoprotection activity. Other designers have had to deviate from the IM steps (Kwak et al., 2007; McEachan et al., 2008). As we are currently testing XPAND, we do not know if the thorough, multi-theory approach justified the time required. However, we recommend that a validated short-form of IM is developed for use in complex behaviours and contexts without adequate time and resources. Bartholomew Eldredge et al. (2016) do suggest that steps can be adapted to fill time available, but more specific adaptions (e.g. use of a theoretical framework rather than multiple individual theories; Heath et al., 2015) are needed, otherwise IM will be beyond the scope of intervention designers working in small teams or targeting complex behaviours in non-academic settings.

### Limitations

Since XP is such a rare disease, we were not able run a pilot study, as there were not enough adults with XP to participate in a pilot and main trial. Despite our efforts to compensate by involvement of the PPI panel and ineligible patients (i.e. those with excellent adherence), this could impact on the feasibility, efficacy, and acceptability of XPAND. This is just one aspect of the broader challenges in operationalising current guidelines for complex intervention development in a rare disease, reported elsewhere (Sainsbury, Walburn, Araujo‐Soares, & Weinman, 2018). Although XPAND did acknowledge that behaviour change occurs in a multilevel social context, it focused on altering the individuals’ responses to social barriers (e.g. lack of social support and stigma) and was not designed to target organisational determinants of UVR exposure (e.g. timing of the working day) that would likely lead to considerable improvements in photoprotection and risk status.

## Conclusion

We have developed a novel, multicomponent intervention, which is tightly bound to theory and personalised at the point of delivery. The mixed-methods approach used in the formative research phase increased confidence that we had a comprehensive understanding of photoprotection and its determinants in XP. Using the systematic approach of IM, we were able to infuse this knowledge into all components of XPAND. We have provided a transparent step-by-step account, which, if XPAND improves photoprotection, can guide an adaptation for other conditions requiring photoprotection.

## Supplementary Material

Supplemental Material
