# Supplementary material for: Why? What? How? Using an Intervention Mapping approach to develop a personalised intervention to improve adherence to photoprotection in patients with Xeroderma Pigmentosum
Source: Health Psychol Behav Med. 2020 Oct 27;8(1):475–500. doi: 10.1080/21642850.2020.1819287 (PMC8114411; doi:10.1080/21642850.2020.1819287)
Supplement: Supplemental Material [file RHPB_A_1819287_SM1561.zip › suppl_data/Supplementary file 3. Evidence statement and Intervention recommendation.docx]

Supplementary file 3.

**An exemplar of an Evidence Statement**

| **EVIDENCE STATEMENT**  **DATA SOURCE:** Cross-sectional International survey (UK, France, Germany, USA)  **STATEMENT:** For the whole sample, total score mean for necessity of photoprotection behaviours was significantly related (p<.05) to UVR protection (OR: 1.95, 95%CI: 1.36-2.77), adherence to face photoprotection on cloudy days (OR: 2.40, 95%CI: 1.72-3.35) and sunny days (OR: 2.47, 95%CI: 1.76-3.47), and body photoprotection on cloudy days (OR: 2.68 95%CI: 1.88-3.81) and sunny days (OR: 2.70, 95%CI: 1.90-3.85). When controlling for demographic and clinical variables, concerns for photoprotection remained significantly related with UVR photoprotection (OR: 1.71, 95%CI: 1.18-2.49), adherence to face photoprotection on cloudy days (OR: 2.07, 95%CI: 1.44-2.97) and sunny days (OR: 2.18, 95%CI 1.50-3.17), and body photoprotection on cloudy days (OR: 2.18, 95%CI: 1.51-3.14) and sunny days (OR: 2.37, 95%CI: 1.62-3.47).  **DETERMINANT:** The determinant is *“necessity of photoprotection”* and is modifiable |
| --- |

**An exemplar of a draft intervention recommendation statement**

**RECOMMENDATION: To improve photoprotection, the intervention should include tools to establish routines and habits.**

**RATIONALE:** A lack of routine associated with photoprotection practices was associated with worse photoprotection of the face and body in the international survey, in both cloudy and sunny conditions (small-to-medium statistical effect sizes). It was also associated with less frequent avoidance of going outside in cloudy weather. Greater automaticity was associated with lower UVR dose to the face (small effect). A lack of routine was characteristic of the adults with poorer protection who were categorised in the qualitative analysis as “resistant to photoprotection” having adjusted to XP in a way that did not impact on their sense of self or everyday life. Intra-individual analysis in the N-of-1 study found that planning (a key strategy to develop a routine) was associated with better photoprotection. Once a habit is established, the amount of conscious planning required will be less.

**NON/MODIFIABLE:** Techniques to develop and maintain habit are well-established in the behaviour change literature (e.g., cues, prompts, reminders in the environment, implementation intentions, behavioural repetition) and could be used in the XP intervention.

**SOURCE OF EVIDENCE:** Qualitative study, N-of-1 study, Dose-to-Face study, Cross-sectional International survey
