# Supplementary material for: Why? What? How? Using an Intervention Mapping approach to develop a personalised intervention to improve adherence to photoprotection in patients with Xeroderma Pigmentosum
Source: Health Psychol Behav Med. 2020 Oct 27;8(1):475–500. doi: 10.1080/21642850.2020.1819287 (PMC8114411; doi:10.1080/21642850.2020.1819287)
Supplement: Supplemental Material [file RHPB_A_1819287_SM1561.zip › suppl_data/Supplementary file 5. Behavioural and Performance Objectives.docx]

**Supplementary file 5. The Behavioural and Performance Objectives**

| **Behavioural objectives** | **Performance objectives** | **Performance objectives** | **Performance objectives** | **Performance**  **objectives** |
| --- | --- | --- | --- | --- |
| 1. Apply the appropriate amount of broad-spectrum factor 50 sunscreen and lip-block, in the correct way, consistently, before going outside | Obtain broad-spectrum factor 50 sunscreen | Make decision to apply the sunscreen and lip-block | Apply the correct amount in the correct way | Maintain sunscreen use every time you go outside |
| 2. Re-apply sunscreen and lip-block every 2 hours | Have sunscreen to hand to allow ease of re-application | Make the decision to re-apply | Re-apply the correct amount, in the correct way, at the correct time | Maintain reapplication |
| 3. Consistently wear a >good combination of photoprotective clothing to protect the face | Have access to photoprotective clothing | Make the decision to wear the clothing combination | Wear the clothing in the most protective way (e.g., buff up to nose) | Maintain wearing adequate combination of photoprotective clothing for face every time you go outside |
| 4. Consistently adjust outside daily activities to reduce UVR exposure within realistic parameters | Decide if daily activities can be adjusted to reduce UVR exposure | Make the decision to adjust activity | Negotiate activities with other people | Continue to adjust activities |
