# Supplementary material for: Why? What? How? Using an Intervention Mapping approach to develop a personalised intervention to improve adherence to photoprotection in patients with Xeroderma Pigmentosum
Source: Health Psychol Behav Med. 2020 Oct 27;8(1):475–500. doi: 10.1080/21642850.2020.1819287 (PMC8114411; doi:10.1080/21642850.2020.1819287)
Supplement: Supplemental Material [file RHPB_A_1819287_SM1561.zip › suppl_data/Supplementary file 7a. CO mapped to BCTS and MOD core content.docx]

Supplementary file 7a. Excerpt from the XPAND master matrix mapping change objectives for each determinant to theory, behaviour-change strategies and modes of delivery: Core content

**Intervention Mode of Delivery**

| **Change Objectives:**  **General and a specific exemplar linked to a photoprotection activity** | **Behaviour-change strategies mapped to taxonomies [Intervention Mapping (IM)** (**Bartholomew Eldredge, Markham, Kok, Ruiter, & Parcel, 2016); Taxonomy of Behaviour Change (Michie et al., 2013)]** | **Key Theory/Framework** | **One-to-one session** | **Magazine** | **Text messages** | **Video showing sunscreen application** | **Other materials**  [Photoprotection goal-setting tool – *UVR Dial*  UVR Photoprotection Feedback sheet Goal-setting record sheet] |
| --- | --- | --- | --- | --- | --- | --- | --- |
| 1. **Photoprotection activities become habitual**   Increase chances that new sunscreen application will become habitual | **Intervention Mapping (IM):** Implementation intentions^1;^ cue altering^1;^ planning coping responses^1^  **BCTv1**: action planning; prompts/cues (7.1); habit formation (8.3); | **TDF** (Goals)  **HT** | Habit formation strategies: (adapted from Gardner, Lally, & Wardle, 2012; Lally & Gardner, 2013) focus on linking photoprotection to existing routines; use of prompts and cues for photoprotection that will trigger them to protect (e.g., hat by the door); explain importance of repeating the activity in the same circumstances.  Facilitator helps participant make if-then statements. Facilitator will elicit participant’s own experiences of habit formation and emphasise that extra effort now will increase chances that new behaviour will become automatic and less burdensome over time.  Manual Core Content: Habit formation | Article including practical tips for habit formation- *“How to make sticking to a UVR routine easier”* | External prompts for new behaviour and messages were developed to reinforce concepts.  *“Putting on your sunscreen at the same time in the same place every morning will help it become habit”* | Shows how to link application within existing morning routine | Goal setting record sheet: includes action and coping plans.  Building blocks of behaviour change graphic shows how photoprotection activities can be developed to become habits. |
| 1. **Promote self-efficacy for photoprotection improvement**   Express confidence in the ability to improve photoprotection activities | **IM:** Verbal persuasion^3^ ; goal setting^3;^  provide opportunities for social comparison^7^  **BCTv1:** Goal setting (1.1); verbal persuasion about capability (15.1); focus on past success (15.3) | **TDF** (Beliefs about capabilities)  **SCT** (self-efficacy) | Facilitator uses the dial as a tool for goal setting by promoting self-efficacy for the new photoprotection activities. The facilitator highlights the success the participant is already achieving, highlights their capabilities to achieve the “tweaks” in photoprotection required. The facilitator guides participant to set initial SMART goal at a level that makes success likely.  Manual Core Content:  Reinforcement of self-efficacy for new photoprotection activities | X | X | X | UVR dial shows how significant improvements to photoprotection can be made by relatively small changes in activities (e.g., adding 1 activity), building on what the participant is already doing. |
| 1. ***Promote self-efficacy for photoprotection in face of barriers**   Express confidence in the ability to apply sunscreen correctly and mange barriers | **IM:** Modelling^2 ;^ reinforcement^2^; feedback^2^; verbal persuasion^3^; self-monitoring of behaviour^3^; planning coping responses^3^; Goal setting^3^ provide opportunities for social comparison.^7^  **BCTv1**: Goal setting (1.1); problem solving (1.2); action planning (1.4); review behaviour goal (1.5); instruction on how to perform a behaviour (4.1); social comparison (6.2); credible source (9.1); verbal persuasion about capability (15.1);  focus on past success (15.3) | **TDF** (Beliefs about capabilities)  **SCT** (self-efficacy) | Explore level of self-efficacy. Collaborative brainstorm on ways to overcome barrier. Participants are directed to video and magazine for examples of other patients overcoming barriers and showing how to apply sunscreen. Facilitator explores participant’s past successes [techniques adapted from (Marks & Allegrante, 2005)], followed by planning ahead for barriers to correct application. Next session goal review reinforces any positive change, attribute success to the participant.  Manual Core Content:  Reinforcement of self-efficacy in the face of barriers to photoprotection activities | Article based on experiences of other patients - *“Solve your sunscreen problems”* | *“Unsure how to apply sunscreen? Check out the XPAND application video for hints and tips”* | Use actors playing people with XP to show correct application of sunscreen, focusing on the skill of application.  Acknowledge putting on sunscreen adequately can be a big task but if it becomes habitual it will require less effort. | X |
| 1. **Build intrinsic motivation to photoprotect**   Express drive to wear more protective clothing | **IM:** Persuasive communication^2^; motivational interviewing^2^; feedback^2;^ provide contingent rewards^3^;anticipated regret^4^; consciousness raising^5^; personalize risk^5^; framing^5^  provide opportunities for social comparison^7^  **BCTv1:** Goal setting (1.1); information about health consequences (5.1); salience of consequences (5.2); monitoring of emotional consequences (5.4); social comparison (6.2); pros and cons (9.2); comparative imagining of future outcomes (9.3); credible source (9.1); self-reward (10.9); | **TDF** (intention; goal; emotion)  **ACT** (valued action)  **NCF** (necessity & concerns)  **SDT** (intrinsic vs extrinsic motivation)  **BBTPE** (positive affect) | The facilitator gives personal feedback about level of photoprotection in the previous year, checks still accurate, and highlights the gap between this and the recommendations of the XP clinical team.  Using the donkey, carrot and stick metaphor (ACT based);participants were encouraged to re-frame their reasons for photoprotecting from negative medical events they were avoiding (e.g., skin cancer - sticks) into positive personal non-medical reasons (e.g., if I stay well by protecting better wearing a face buff this will mean I am able to travel more when I retire-carrots). The stick and carrot increase perceived necessity and motivation to photoprotect [Adapted from (Stoddard & Afari, 2014)], Personal reasons were linked to positive emotions which fuel motivation. The importance of positive emotions to drive behaviour change was emphasised (Van Cappellen, Rice, Catalino, & Fredrickson, 2018)  The facilitator asks the participant to set a reward if the SMART goal is achieved.  Manual Core Content:  Build intrinsic motivation to photoprotect | Two stories from people with XP explain what motivates them.  One article *“Reboot your motivation”* describing how to identify personal reasons for protecting and links these to values. | *“Unsure whether to protect today? Take a moment to remind yourself why protecting is important for you”* | X | Feedback sheet showing levels of photoprotection provided by different combinations of clothing (Daily Photoprotection Scale)(Sainsbury et al., 2018) and participants’ own protection level .  Scales graphic showing stick and carrots as reasons why protection is necessary balanced against concerns about photoprotection.  Activity sheet for face to face session 1 (assessment pro forma) with space to record personal reasons for protection. It also included space for a reward. |
| 1. **Plug gaps in knowledge about photoprotection****   Know how to photoprotect the face using clothing | **IM:** Using Imagery^6^  **BCT V1:** Instruction on how to perform the behaviour (4.1) | **TDF** (knowledge) | Facilitator explores participant’s understanding about clothing options (e.g., type of hat, glasses..etc). Directs participant to relevant pages in magazine.  Manual Core Content:  How to photoprotect | Article describing how to protect using clothing *“Look good, feel good*” | *“Uncertain what is the best hat to use for protection? Wear one with 3-inch brim to protect head, neck and ears”* | Video shows character wearing clothing to protect the face. | UVR dial and face protection guide show recommendations from clinical team on what is the optimal level of protection. |
| 1. **Promote self-regulation for new photoprotection activities**   Active use of self-regulation tools to increase frequency of sunscreen application | **IM**: Implementation intentions^1;^ planning coping responses^1,3^; facilitation^1^; self-monitoring of behaviour^2;^ feedback^2^; goal setting^3^; provide contingent rewards^3^  **BCT v1**: Goal setting (1.1); problem solving (1.2); action planning (1.4); review behaviour goal (1.5); social support (practical; 3.2); monitoring of emotional consequences (5.4); pros and cons (9.2); self-reward (10.9); | **TDF**: (goals; behavioural regulation)  **SCT:** (environmental factors)  **BBTPE**: (positive affect) | Facilitator guides participant to set a SMART photoprotection goal e.g., *“I will apply sunscreen 3 times in the next 7 days”*  And an action plan showing the steps needed to occur to enact the behaviour (when/where/how), *who* will support them and how will they reward themselves. Barriers to goal achievement are considered, followed by problem solving and coping planning (“if-then”). Participant is encouraged to monitor whether they achieve the goal by recording progress and devise reward. Goal is reviewed at the next session and positive emotional impacts (e.g., happy, safe, in control, relaxed) are amplified (see also row 3.)  Manual Core Content:  Promote self-regulation for new photoprotection activities (including problem-solving, SMART goal setting, action and coping-planning) | Article describing SMART Goal setting *“Be SMART about your UVR protection”* | *“Achieve your UVR protection goal – plan when where and how!”* | X | Activity sheets [goal setting sheet including action and coping planning adapted from NHS Health trainer handbook (Michie et al., 2008); a volitional help sheet adapted from (Armitage & Arden, 2010)); problem solving sheet adapted from Getselfhelp.com] |
| **Maintenance of positive change**  Continue to wear a face buff when outdoors in the long term | IM: Implementation intentions^1;^ planning coping responses^1,3^; facilitation^1^; self-monitoring of behaviour^2;^ feedback^2^; goal setting^3^;verbal persuasion^3;^ provide contingent rewards.^3.^  **BCT v1**: Goal setting (1.1); problem solving (1.2); action planning (1.4); review behaviour goal (1.5); social support (practical; 3.2); monitoring of emotional consequences (5.4); self reward (10.9); reducing negative emotions (11.2); conserving mental resources (11.3); verbal persuasion about capability (15.1);  focus on past success (15.3) | **TDF:** (goals; behavioural regulation)  **MT**: (motives; self-regulation; resources; habit; environmental and social influences)  **BBTPE:** (positive affect). | During sessions 6 & 7 the facilitator will switch focus to maintenance. This will involve how to maintain increased motivation, keep practicing self-regulatory skills and boosting psychological resources to photoprotect by managing stress and experiencing positive emotions. It is anticipated that establishing habits (row 1) and involving significant others in photoprotection (row 6) will support maintenance. Relapse prevention and managing setbacks is included with a focus on realistic process of behaviour change. Self-efficacy and self- regulatory strategies (rows 3, 6) are applied to the future (e.g., confidence to keep wearing face-buff; coping planning for future barriers to wearing a face-buff).  Manual Core Content:  Support maintenance of positive change | Article detailing key maintenance strategies - “Keep up the good work!” | X | X | Graphic showing trajectory of realistic behaviour change (Michie et al., 2008) |

*Strengthening self-efficacy was core content, however when it was applied in the context of barriers – the barriers themselves were dependent on the individual (i.e. personalised).

**Confirming correct knowledge of gold standard of photoprotection was core content, however gaps in knowledge were personalised.

See table 4b for a description of the personalised content of XPAND

| **ACT** Acceptance and Commitment Therapy (Hayes et al., 2006)  **BBTE** Broaden and Build theory of positive emotions applied to healthcare (Van Cappellen, Rice, Catalino, & Fredrickson, 2018)  **HT** Habit Theory (Verplanken, 2006; Verplanken & Aarts, 1999; Verplanken & Orbell, 2003)  **MT** Multiple theories relevant to maintenance (Kwasnicka, Dombrowski, White, & Sniehotta, 2016)  **TDF** Theoretical Domains Framework (Cane, O’Connor, & Michie, 2012)  **SCT** Social Cognitive Theory (Bandura, 2001)  **SDT** Self Determination Theory (Deci & Ryan, 2000) | Intervention Mapping evidenced-based change methods (Bartholomew Eldredge et al., 2016)  ^1^Methods to change Habitual, Automatic, and Impulsive Behaviors  ^2^Basic methods at the individual level  ^3^Methods to change skills, capability, and self-efficacy and to overcome barriers  ^4^Methods to change attitudes, beliefs and outcome expectations  ^5^Methods to change awareness and risk perception  ^6^Methods to increase knowledge  ^7^ Methods to change social influence |
| --- | --- |

Armitage, C. J., & Arden, M. A. (2010). A volitional help sheet to increase physical activity in people with low socioeconomic status: a randomised exploratory trial. *Psychology and health, 25*(10), 1129-1145. <https://doi.org/10.1080/08870440903121638>

Bandura, A. (2001). Social Cognitive Theory: An Agentic Perspective. *Annual Review of Psychology, 52*(1), 1-26. doi:10.1146/annurev.psych.52.1.1

Cane, J., O’Connor, D., & Michie, S. (2012). Validation of the theoretical domains framework for use in behaviour change and implementation research. *Implementation science, 7*(1), 37. <https://doi.org/10.1186/1748-5908-7-37>

Deci, E. L., & Ryan, R. M. (2000). The" what" and" why" of goal pursuits: Human needs and the self-determination of behavior. *Psychological Inquiry, 11*(4), 227-268. https://doi.org/10.1207/S15327965PLI1104_01

Bartholomew Eldredge, L. K., Markham, C. M., Kok, G., Ruiter, R. A., & Parcel, G. S. (2016). *Planning health promotion programs: an intervention mapping approach* (4^th^

Edition). San Franciso: John Wiley & Sons

Gardner, B., Lally, P., & Wardle, J. (2012). Making health habitual: the psychology of ‘habit-formation’ and general practice*. British Journal of General Practice*

*62*(605), 664-666. doi:10.3399/bjgp12X659466

Hayes, S. C., Luoma, J. B., Bond, F. W., Masuda, A., & Lillis, J. (2006). Acceptance and commitment therapy: Model, processes and outcomes. *Behaviour research and therapy*, *44*(1), 1-25. <https://doi.org/10.1016/j.brat.2005.06.006>

Kwasnicka, D., Dombrowski, S. U., White, M., & Sniehotta, F. (2016). Theoretical explanations for maintenance of behaviour change: a systematic review of behaviour theories. *Health psychology review, 10*(3), 277-296. <https://doi.org/10.1080/17437199.2016.1151372>

Lally, P., & Gardner, B. (2013). Promoting habit formation. *Health Psychology Review, 7*(sup1), S137-S158. <https://doi.org/10.3399/bjgp12X659466>

Marks, R., & Allegrante, J. P. J. H. p. p. (2005). A review and synthesis of research evidence for self-efficacy-enhancing interventions for reducing chronic disability: implications for health education practice (part II). *Health promotion practice*, *6*(2), 148-156. [https://doi.org/10.1177/1524839904266792](https://doi.org/10.1177%2F1524839904266792)

Michie, S., Richardson, M., Johnston, M., Abraham, C., Francis, J., Hardeman, W., . . . Wood, C. E. (2013). The behavior change technique taxonomy (v1) of 93 hierarchically clustered techniques: building an international consensus for the reporting of behavior change interventions. *Annals of Behavioral Medicine, 46*(1), 81-95. <https://doi.org/10.1007/s12160-013-9486-6>

Michie, S., Rumsey, N., Fussell, A., Hardeman, W., Johnston, M., Newman, S., & Yardley, L. (2008). Improving health: changing behaviour. NHS health trainer handbook. In: Department of Health Publications (Best Practice Guidance: Gateway Ref 9721).

Sainsbury, K., Vieira, R., Walburn, J., Sniehotta, F. F., Sarkany, R., Weinman, J., & Araujo-Soares, V. (2018). Understanding and predicting a complex behaviour using n-of-methods: Photoprotection in xeroderma pigmentosum. *Health Psychology*. *37*(12), 1145–1158. [https://doi.org/10.1037/hea0000673](https://psycnet.apa.org/doi/10.1037/hea0000673)

Stoddard, J. A., & Afari, N. (2014). *The Big Book of ACT Metaphors: a practitioner's guide to experiential exercises and metaphors in Acceptance and Commitment Therapy*. Oakland, CA: New Harbinger Publications.

Van Cappellen, P., Rice, E. L., Catalino, L. I., & Fredrickson, B. L. (2018). Positive affective processes underlie positive health behaviour change. *Psychology & Health, 33*(1), 77-97. <https://doi.org/10.1080/08870446.2017.1320798>

Verplanken, B. (2006). Beyond frequency: Habit as mental construct. *British Journal of Social Psychology,* 45(3), 639-656*.* doi:10.1348/014466605x49122

Verplanken, B., & Aarts, H. (1999). Habit, attitude, and planned behaviour: Is habit an empty construct or an interesting case of goal-directed automaticity? *European review of social psychology*, *10*(1), 101-134. <https://doi.org/10.1080/14792779943000035>

Verplanken, B., & Orbell, S. (2003). Reflections on past behavior: A self-report index of habit strength. *Journal of applied social psychology*, *33*(6), 1313-1330. <https://doi.org/10.1111/j.1559-1816.2003.tb01951.x>
