# Supplementary material for: Why? What? How? Using an Intervention Mapping approach to develop a personalised intervention to improve adherence to photoprotection in patients with Xeroderma Pigmentosum
Source: Health Psychol Behav Med. 2020 Oct 27;8(1):475–500. doi: 10.1080/21642850.2020.1819287 (PMC8114411; doi:10.1080/21642850.2020.1819287)
Supplement: Supplemental Material [file RHPB_A_1819287_SM1561.zip › suppl_data/Supplementary file 7b. CO mapped to BCTs and MOD personalised content.docx]

Supplementary file 7b. Excerpt from the XPAND master matrix mapping change objectives for each determinant to theory, behaviour-change strategies and modes of delivery: Personalised content

**Intervention Mode of Delivery**

| **Change Objectives: General and a specific exemplar linked to a photoprotection activity** | **Behaviour-change strategies mapped to taxonomies [Intervention Mapping (Eldredge, Markham, Kok, Ruiter, & Parcel, 2016); Taxonomy of Behaviour Change (Michie et al., 2013)]** | **Key Theory or Framework** | **One-to-one session**  **(Summary of content included in the intervention manual)** | **Magazine** | **Text messages** | **Video showing sunscreen application** | **Other materials**  [Determinant-specific activity sheets] |
| --- | --- | --- | --- | --- | --- | --- | --- |
| 1. **Strengthen perception of the need to photoprotect***   Express strong belief in UVR risk regardless of weather, season (heat/sun/clouds) or time of day. | **Intervention Mapping (IM):** Arguments^,4^; Persuasive communication^2^, belief selection^2^, tailoring^2^, modelling ^2^; reinforcement ^2 ;^ consciousness raising^3^; personalize risk^5^, scenario risk based information^5^; using imagery^6^; provide opportunities for social comparison^7^  **BCTv1:** Information about health consequences (5.1); Salience of consequences (5.2); Information about social and environmental consequences (5.3);  social comparison (6.2); credible source (9.1); pros and cons (9.2); incompatible beliefs (13.3) | **TDF**  (beliefs about consequences, knowledge)  **NCF** (necessity) | Aim is to increase strength of belief in the necessity to use photoprotection. It challenges misunderstandings about photoprotection and look for gaps in knowledge that are driving low necessity beliefs. The facilitator elicits and normalises personal doubts and experiences (e.g., skin cancer despite protecting) using stories from other patients, experts, and seek permission to provide tailored evidence about levels of UVR in different environmental conditions. The facilitator explains that UVR damage is cumulative and cannot be reversed.  Manual module/s: Perceived necessity of photoprotection | Two articles explaining how UVR is present if there is daylight across different weathers and seasons – *“10 UVR myth busters”; “The truth about your environment”*  A patient story emphasising that risk is present if there is daylight.  Visual of car’s mileometer to show how damage is cumulative and non-reversable. | *UV rays are invisible… don’t let that fool you! Protect if there is daylight - whatever the weather or time of day* | Audio reinforced need to protect in all weathers | X |
| 1. **Reduce practical concerns about photoprotection****   Express fewer concerns about using sunscreen | **IM:** belief selection^2^; tailoring^2^; modelling ^2^; planning coping responses^2^; reinforcement^2^; provide opportunities for social comparison^7^  **BCTv1:** Problem solving (1.2); instruction on how to perform the behaviour (4.1); social comparison (6.2) | **TDF**  (beliefs about consequences, knowledge, skills)  **NCF** (concerns) | The aim is to minimise concerns about using sunscreen. The conversation is tailored to the specific concern of the individual. The facilitator normalises concerns about sunscreen and obstacles to using it. This involves investigation of impact of different contexts (environmental, activity, social) on barrier and whether it can be modified. The balance of the concerns versus the strength of necessity to apply sunscreen is discussed.  Manual module/s:  Core-content: Problem solving | Article focused on sunscreen concerns *“Solve your sunscreen problems”.* It  acknowledges that concerns about treatment regime are normal and includes barriers from other patients. | *“If your sunscreen is too sticky, check out other types. Contact the XP team for more advice”* | Does not focus on concerns but video of correct sunscreen application could inadvertently be relevant to the concern of the individual. | Problem solving activity sheet adapted from Getselfhelp.com |
| 1. **Reduce concerns about looking different whilst photoprotecting**   Express fewer concerns about others' reactions to wearing a face-buff | **IM:** belief selection^2^; tailoring^2^; modelling ^2^; planning coping responses^2^; persuasive communication^2^; reinforcement^2^; self- monitoring of behaviour^3^; reattribution training^3^; provide opportunities for social comparison^7^  **BCTv1:** Problem solving (1.2); instruction on how to perform the behaviour (4.1); reattribution (4.3); behavioural experiments (4.4); Information about emotional consequences (5.6); social comparison (6.2); credible source (9.1); pros and cons (9.2) | **TDF**  (beliefs about consequences; skills)  **NCF** (concerns)  **CBT**  (attention training; social skills and coping strategies) | The aim is to affirm appearance concerns and acknowledge that they can be an important part of the daily burden of having XP. It provides practical strategies to manage unwanted attention involving diversion of attention in the moment, choosing types of protection that are more likely to blend in and boosts general social skills. Content adapted from existing manual (Clarke, Thompson, Jenkinson, Rumsey, & Newell, 2013).  Manual module/s:  Concerns about appearance when photoprotecting | Article on managing barriers to photoprotection includes key strategies to manage appearance worries – *“What’s stopping you getting the UVR protection you need?”* | X | X | Activity sheet reiterates that concerns about appearance are natural; summarises tips to manage staring; gives examples relevant to photoprotection. |
| 1. **Promote acceptance of XP and photoprotection**   Express acceptance of wearing UVR glasses in all weathers | **IM:** Anticipated regret^1^;  active learning^2;^ reinforcement^2^ ; persuasive communication^2^;  self-re-evaluation^3^; dramatic Relief^3;^ self affirmation^3^ ;  **BCTv1:** Salience of consequences (5.2) Information about social and environmental consequences (5.3)  Monitoring of emotional consequences (5.4)  Anticipated regret (5.5)  Information about emotional consequences (5.6); comparative imagining of future outcomes (9.3);  framing/reframing (13.2);  incompatible beliefs (13.3 ); valued self-identity (13.4)Self-talk (15.4) | **ACT**  (values, discrepancy between behaviour and long-term values;  willingness and acceptance) | These build on core motivation content and clarify personal values; explore how current photoprotection fits with these values; encourage the identification of discrepancies between current level of UVR glasses and values (in the longer-term); discussion around the concepts of acceptance and willingness; and providing strategies to minimise discrepancies so that wearing glasses can be considered as a way of acting in line with values (vs. getting in the way of valued living) [Adapted from (Harris, 2009; Stoddard & Afari, 2014)].  Manual module/s:  Values and photoprotection  Willingness and photoprotection | One article *“Reboot your motivation”* describing how to identify personal reasons for protecting and links these to values. | *XP is just one part of you – protect well so you can keep doing all the other things that make you who you are* | X | Activity sheets reinforcing concepts of values and willingness. Lists of values and tips for how to use them to fuel motivation to photoprotect, are included. Active nature of willingness and acceptance is highlighted. |
| 1. **Facilitate mobilisation of helpful support for photoprotection**   Express motivation to elicit support of family and friends in relation to wearing a hat and glasses in all weathers | **IM:** tailoring^2^; modelling ^2^ ; planning coping responses^2^ ; reinforcement^2^; self- monitoring of behaviour^3^; mobilising social resources^7^; provide opportunities for social comparison.^7^  **BCTv1:** Problem solving (1.2); social support (practical) (3.2); social support (emotional) (3.3); Instruction on how to perform the behaviour (4.1); behavioural experiments (4.4); Information about emotional consequences (5.6); social comparison (6.2); credible source (9.1); pros and cons (9.2). | **TDF** (social influences; beliefs about consequences; skills). | The aim is to encourage people to utilize the support that they have available, to promote better adherence. It focuses on functional rather than structural support, and the direct effect on health (not only as a stress-buffer). It explores the perception of support and highlights that support can be unhelpful. It primarily targets practical support. It includes the importance of disclosure as a key step to receive the support that is most useful for the person. Communication skills are discussed.  Manual module/s:  Mobilisation of social support and disclosure | One article highlights how family and friends can become involved in photoprotection. It describes emotional and practical support, and encourages the reader to think about their own support network – *“Your support network”*  One article discusses the pros and cons of disclosing in order to receive support  *“Talking about XP*”  Patient story describing experiences of helpful support . | *Get the right help for your UVR protection. Think about what would work best for you and tell your friends about it.* | X | Activity sheet reinforces skills and concepts discussed in the session. Summary of key communication skills adapted from (Sage, Sowden, Chorlton, & Edeleanu, 2013). |
| 1. **Minimise impact of positive or negative emotions that reduce photoprotection**   Recognise impact of emotions in-the- moment and background low mood on reapplying sunscreen | tailoring^2^; planning coping responses^2^; reinforcement^2^; self- monitoring of behaviour^3^; Improving physical and emotional states ^3^ ; Anticipated regret.^4^  **BCTv1:**  Problem solving (1.2); Monitoring of emotional consequences (5.4); Anticipated regret (5.5); Information about emotional consequences (5.6); Pros and cons (9.2); Reducing negative emotion (11.2) | **TDF** (Skills; Emotions)  Transactional model of stress and coping  **CBT**  (stress management) | The aim is to modify emotion if it has a negative impact on photoprotection. The relationship between emotions (positive and negative) and photoprotection will be explored (i.e., not wishing to wear face-buff when feeling happy in case it lowers mood). Facilitators will provide cognitive, emotional, and behavioural strategies to manage fluctuations in mood and stress, to minimise influence on protection and improve emotional stability in the long-term.  Manual module/s:  Mood and photoprotection  Stress and photoprotection | Article on managing barriers to photoprotection includes positive and negative emotions as a barrier – *“What’s stopping you getting the UVR protection you need?”* | *Feeling good today and don’t want protection to bring you down? Remind yourself how protecting now can help you to achieve the things you want in the future.* | X | Activity sheets reinforce skills and concepts discussed in the session. Content related to pleasant activity scheduling adapted from Getselfhelp.co.uk. Symptoms of low mood adapted from https://www.nhs.uk/conditions/stress-anxiety-depression/low-mood-and-depression/ |

*Content addressed different perceptions that contributed to doubts about necessity of photoprotection - underestimation of risk of UVR exposure in different environmental conditions and duration outdoors; underestimation of skin cancer risk; fatalistic beliefs related to cancer; underestimation of risk if UVR exposure does not result in sunburn; overestimation of the effectiveness of clinical treatment and underestimation of self-management; doubts about the effectiveness of photoprotection. The table includes one example.

** Problem solving as a skill was considered relevant to all, so was included in the manual as core content. It is included here, as it was the strategy used to address practical concerns, which was only delivered if the participant had such concerns.

**CBT** Cognitive Behavioural Therapy

Intervention Mapping evidenced-based change methods (Bartholomew Eldredge et al., 2016)

^1^Methods to change Habitual, Automatic, and Impulsive Behaviors

^2^Basic methods at the individual level

^3^Methods to change skills, capability, and self-efficacy and to overcome barriers

^4^Methods to change attitudes, beliefs and outcome expectations

^5^Methods to change awareness and risk perception

^6^Methods to increase knowledge

^7^ Methods to change social influence

Clarke, A., Thompson, A. R., Jenkinson, E., Rumsey, N., & Newell, R. (2013). *CBT for appearance anxiety: psychosocial interventions for anxiety due to visible difference*. West Sussex, UK: Wiley-Blackwell

Bartholomew Eldredge, L. K., Markham, C. M., Kok, G., Ruiter, R. A., & Parcel, G. S. (2016). *Planning health promotion programs: an intervention mapping approach* (4^th^ Edition). San Franciso: John Wiley & Sons.

Harris, R. (2009). *ACT made simple: An easy-to-read primer on acceptance and commitment therapy*. Oakland CA: New Harbinger Publications.

Michie, S., Richardson, M., Johnston, M., Abraham, C., Francis, J., Hardeman, W., . . . Wood, C. E. (2013). The behavior change technique taxonomy (v1) of 93 hierarchically clustered techniques: building an international consensus for the reporting of behavior change interventions. *Annals of Behavioral Medicine, 46*(1), 81-95. <https://doi.org/10.1007/s12160-013-9486-6>

Sage, N., Sowden, M., Chorlton, E., & Edeleanu, A. (2013). *CBT for chronic illness and palliative care: A workbook and toolkit*. Chichester: John Wiley & Sons.

Stoddard, J. A., & Afari, N. (2014). *The Big Book of ACT Metaphors: a practitioner's guide to experiential exercises and metaphors in Acceptance and Commitment Therapy*. Oakland, CA: New Harbinger Publications.
