# Supplementary material for: Why? What? How? Using an Intervention Mapping approach to develop a personalised intervention to improve adherence to photoprotection in patients with Xeroderma Pigmentosum
Source: Health Psychol Behav Med. 2020 Oct 27;8(1):475–500. doi: 10.1080/21642850.2020.1819287 (PMC8114411; doi:10.1080/21642850.2020.1819287)
Supplement: Supplemental Material [file RHPB_A_1819287_SM1561.zip › suppl_data/Supplementary file 8 XPAND text bank 04.02.2020.docx]

Supplementary file 8. XPAND text bank

| **DRIVER** | **TEXT MESSAGE** | **REFERENCE (if adapted from elsewhere)** |
| --- | --- | --- |
| **Low necessity** |  |  |
| 1. Underestimation of risk | It’s important to protect from UVR - whatever the weather |  |
| 1. Underestimation of risk | UV rays are invisible… don’t let that fool you! Protect whatever the weather, or time of day | (Petrie, Perry, Broadbent, & Weinman, 2012) |
| 1. Underestimation of risk | You cannot always see sun damage but it doesn’t mean it’s not there. Always protect. | (Petrie et al., 2012) |
| 1. Underestimation of risk | Reduce your risk of XP complications by always maintaining high levels of UVR protection |  |
| 1. Doubts about effectiveness of UVR   protection | Sunscreen and good clothing protection lower your chances of skin damage. Don’t forget yours! |  |
| 1. Doubts about effectiveness of UVR protection | Unprotected UVR exposure will increase your cancer risk. Don’t take the chance – apply sunscreen daily |  |
| 1. Low personal control | Everyone is at risk from UVR damage and you have the knowledge to reduce your personal risk – keep at it! |  |
| 1. Low personal control | Make sure you control your XP by always protecting against UVR. You’re the boss! | (Petrie et al., 2012) |
| 1. Extreme treatment control | Keeping your skin healthy is a team effort – you protect from UVR and the XP service monitor and treat |  |
| 1. Doubts about effectiveness of UVR protection | A good base layer of factor 50+ in the morning makes sense – don’t skip it! Sunscreen, clothing and SMART scheduling lower your risk of skin damage |  |
| **High concerns about protection – focus sunscreen** |  |  |
| 1. Sunscreen application | Let your sunscreen absorb properly before rushing out – invest in your health |  |
| 1. Sunscreen application | Sunscreens with SPF wear off during the day because of sweat, oils, light rays, and routine skin exposure. Remember to reapply regularly |  |
| 1. Sunscreen application | If your sunscreen is too sticky, check out other types. Contact the XP team for more advice |  |
| 1. Sunscreen application | Stay smart, stay UV safe. Set your phone to remind you to reapply sunscreen |  |
| 1. Sunscreen application | It won’t work if it’s still in the bottle! Apply your sunscreen regularly and generously. |  |
| 1. Sunscreen   Application | Sunscreen works best when applied generously. Use 1 teaspoon to cover your face and neck. |  |
| 1. Clothing | Feel too hot covering up? Check out XPAND magazine for hints and tips on clothing options |  |
| 1. Sunscreen application | Don’t forget to take your UV meter, spare sunscreen, lip balm out with you today |  |
| 1. Clothing | Unsure whether you want to wear dark glasses? Clear glasses can also protect your eyes from UVR damage. Check with your optician. |  |
| 1. Sunscreen application | Others with XP say it can be boring and repetitive to apply sunscreen. It’s worth it, though, and so are you! |  |
| **Positive or negative emotions that reduce protection** |  |  |
| 1. Emotions in the moment | Feeling good today and don’t want protection to bring you down? Remind yourself how protecting now can help you to achieve the things you want in the future. |  |
| 1. Emotions in the moment | Feeling stressed? We all feel pressured from time to time. Make plans to see people you know make you feel better. |  |
| 1. Emotions in the moment | Feeling low on energy? Make sure you fill-up your tank by doing something you enjoy. |  |
| 1. Emotions in the moment | Boost your motivation to protect - check out XPAND magazine for hints and tips |  |
| 1. Emotions in the moment | Tempted not to protect? Avoid feeling worried later by protecting your skin today |  |
| 1. Low mood | Feeling fed-up? Plan to do something you enjoy. Treat yourself! Recognise all the effort you put in to protect yourself from UVR. |  |
| 1. Low mood prevention | Keep your motivation to protect topped up – Read about how other people with XP keep motivated in the XPAND magazine |  |
| 1. Low mood prevention | Protect your emotional wellbeing – look after yourself - eat well, keep physically active and get enough sleep |  |
| 1. Emotions in the moment | Tempted not to protect? Talk to your family or friends - let them encourage you. |  |
| 1. Low mood | Feeling low? Treat yourself! Recognise all the effort you put in to protect yourself from UVR. |  |
| **Poor mobilization of helpful social support, stigma and social norms** |  |  |
| 1. Social norms | Remind someone today of the fact that everyone needs to protect themselves from UV damage, not just you. |  |
| 1. Poor mobilization of support | Everyone needs friends – think about who you can help today and who could help you |  |
| 1. Toxic social context | Seek out situations where you feel nourished, not side-lined. You have a lot to contribute to life. |  |
| 1. Poor mobilization of support | Sharing a challenge with a friend can keep you motivated. Can you ask someone to support you? | (Janda, Youl, Marshall, Soyer, & Baade, 2013) |
| 1. Poor mobilization of support | Get the right help for your UVR protection. Think about what would work best for you and tell your friends about it. |  |
| 1. Poor mobilization of support | Reminders? Taking on outdoor chores? Shifting plans to the evening? Whatever helps you to protect – Get others involved |  |
| 1. Toxic social context | Your health is the most important thing today, and every day, don’t allow others to blow you off course |  |
| 1. Poor mobilization of support | Help others to understand why you need to protect – show them the XPAND magazine |  |
| 1. Poor mobilization of support | Don’t feel you have someone you can talk to about XP? Check out the XP support group http://xpsupportgroup.org.uk/ and Teddington trust http://www.teddingtontrust.com/ |  |
| 1. Poor mobilization of support | Gain a different perspective - share your photoprotection worries with someone you trust |  |
| **Importance of non-health priorities, balance and feeling different** |  |  |
| 1. Importance of non-health priorities | Life makes constant demands but health must be a priority. Protect well today |  |
| 1. Importance of non-health priorities | How many balls are you juggling today? Remember the vital one – your UVR protection! |  |
| 1. Avoid difference | Difference is what makes life exciting. Embrace yours! |  |
| 1. Avoid difference | Everyone has personal/ health issues- you can reduce your risks of skin damage with good UVR protection |  |
| 1. Importance of non-health priorities | It is always worth the extra time and effort to UV protect – make yourself and protection a priority |  |
| 1. Importance of non-health priorities | Unsure whether to protect today? Take a moment to remind yourself why protecting is important for you. |  |
| 1. Importance of non-health priorities | JUST DO IT! You know what I’m talking about! |  |
| 1. Balance | XP is just one part of you – protect well so you can keep doing all the other things that make you who you are |  |
| 1. Importance of non-health priorities | Make time to protect well today – keeping healthy is important – it makes other things possible |  |
| 1. Importance of non-health priorities | Clean the house, put washing on, pick up children, go to work, call your friends, shopping - life is busy. Don’t forget your UVR protection! |  |
| **Low self-efficacy/in presence of barriers** |  |  |
| 1. Low self-efficacy | By applying generous amounts of sunscreen as a daily routine, your confidence will grow. Do it every day |  |
| 1. Low self-efficacy in presence of barriers | Life constantly throws us challenges and you have the strength to meet them. Always protect from UVR |  |
| 1. Low self-efficacy | Unsure how to apply sunscreen? Check out the XPAND application video for hints and tips |  |
| 1. Low self-efficacy in presence of barriers | Uncertain what is the best hat to use for protection? Wear one with 3-inch brim to protect head, neck and ears. |  |
| 1. Low self-efficacy in presence of barriers | Facing an unexpected protection challenge? Think about how you’ve coped before - you have the skills to deal with it. |  |
| 1. Low self-efficacy in presence of barriers | Is something interfering with your protection? Check out how other people with XP have coped in the XPAND magazine |  |
| 1. Low self-efficacy in presence of barriers | Unsure how you’ll keep up your protection on holiday? Plan ahead so you can do what you enjoy whilst protecting your skin |  |
| 1. Low self-efficacy in presence of barriers | Facing an unexpected protection challenge? Think about how your friends and family can support you to work it out |  |
| 1. Low self-efficacy in presence of barriers | Feeling unsure about how to talk to someone new about photoprotection? Practice what you will say with someone you trust. |  |
| 10. Low self-efficacy in presence of barriers | Consistently dealing with obstacles to protection will increase your confidence that you can protect well no matter what. |  |
| **Poor self-regulation + habit formation** |  |  |
| 1. Poor self-regulation | Make today your ‘organise my UVR protection’ day – sort out reminders/ people/ places/ timings/ things that help. |  |
| 1. Habit formation | Too much effort to apply sunscreen every morning? Link to your morning routine and it will soon be less demanding |  |
| 1. Habit formation | Putting on your sunscreen at the *same time* in the *same place* every morning will help it become habit |  |
| 1. Poor self-regulation | Achieve your UVR protection goal – plan when where and how! |  |
| 1. Habit formation | Give yourself the best chance – keep your sunscreen bottle and UVR clothing where you can see them |  |
| 1. Habit formation | Remember, a little effort now = much less effort in the long term. |  |
| 1. Poor self- regulation | Try not to feel bad if you don’t quite reach your goal, changing behaviour is not easy. Try again tomorrow…. |  |
| 1. Poor self- regulation | Plan ahead for challenges – they are less likely to get in the way of your UVR protection |  |
| 1. Poor self-regulation | Plan your day – can you adjust timings or activities so they lower your UVR exposure? |  |
| 1. Poor self-regulation | What is your reward for success this week? Rewards help to re-fuel motivation – choose something that is meaningful for you. |  |
| **KNOWLEDGE + EXTRAS** |  |  |
| 1. Self-regulation | At times, daily routines are hard. Keep positive – you are definitely worth it! |  |
| 1. Knowledge | UVR is energy emitted from the sun. It causes sun damage, wrinkles, and more. Protect yourself and remind others they need to too. | (Hingle et al., 2014) |
| 1. Knowledge | Sunscreens with SPF wear off during the day because of sweat, oils, light rays, and routine skin exposure. Remember to reapply regularly | (Hingle et al., 2014) |
| 1. Knowledge | Your skin is thinnest on the eyelid. Cover delicate areas to protect them and don’t forget to apply, and reapply sunscreen to your face including eyelids, and lips. | (Hingle et al., 2014) |
| 1. Knowledge | Reduce your risk of XP complications by always maintaining high levels of UVR protection |  |
| 1. Doubts about effectiveness of UVR protection | Unprotected UVR exposure will increase your cancer risk. Don’t take the chance – wear your protective clothing today |  |

Hingle, M. D., Snyder, A. L., McKenzie, N. E., Thomson, C. A., Logan, R. A., Ellison, E. A., . . . Harris, R. B. J. A. j. o. p. m. (2014). Effects of a short messaging service–based skin cancer prevention campaign in adolescents. *47*(5), 617-623.

Janda, M., Youl, P., Marshall, A. L., Soyer, H., & Baade, P. J. C. c. t. (2013). The HealthyTexts study: A randomized controlled trial to improve skin cancer prevention behaviors among young people. *35*(1), 159-167.

Petrie, K. J., Perry, K., Broadbent, E., & Weinman, J. (2012). A text message programme designed to modify patients' illness and treatment beliefs improves self-reported adherence to asthma preventer medication. *British Journal of Health Psychology, 17*(1), 74-84. doi:10.1111/j.2044-8287.2011.02033.x
