# Supplementary material for: Why? What? How? Using an Intervention Mapping approach to develop a personalised intervention to improve adherence to photoprotection in patients with Xeroderma Pigmentosum
Source: Health Psychol Behav Med. 2020 Oct 27;8(1):475–500. doi: 10.1080/21642850.2020.1819287 (PMC8114411; doi:10.1080/21642850.2020.1819287)
Supplement: Supplemental Material [file RHPB_A_1819287_SM1561.zip › suppl_data/Supplementary_file_6._Change_Objectives_for_sunscreen_PO-clean.docx]

|  | **Necessity beliefs about photoprotection**  Risk perception  XP and Cancer | **Necessity beliefs about photoprotection**  Risk perception  Environmental conditions | **Necessity beliefs about photoprotection**  Effectiveness of photoprotection | **Concerns about photoprotection** | **Self-efficacy** | **Prioritisation of photoprotection** | **Motivation** | **Emotion** |
| --- | --- | --- | --- | --- | --- | --- | --- | --- |
| **Performance Objective**  Make decision to apply the sunscreen and lip-block | **1.** Express strong belief in own risk of skin cancer due to XP (describe how XP works in relation to UVR damage).  **2**. Express strong belief in the link between UVR exposure and skin cancer. | **1**. Express strong belief in UVR risk regardless of season.  **2.** Express strong belief in UVR risk regardless of weather  (heat/sun/clouds).  **3.** Express strong belief in UVR risk regardless of time of day. | **1.** Express strong belief in the effectiveness of sunscreen to protect against UVR.  **2**. Express strong belief in efficacy of sunscreen to protect against cancer.  **NOTE**: make sure this conversation includes all combinations of photoprotection activities, so the participant doesn't come to the conclusion that sunscreen is sufficient, also need other photoprotection activities. | **1.** Recognise own concerns about using sunscreen.  **2.** Have fewer unrealistic concerns about using sunscreen. | **1.** Express confidence in the ability to apply sunscreen in the presence of barriers. | **1.** Acknowledge the diagnosis of XP.  **2**. Align photoprotection requirements with view of self.  **3**. Align photoprotection requirements with personal reasons for photoprotection and life values.  **4.** Recognise that the application of sunscreen is not in conflict with own "non-XP" identity | **1.** Express a drive to apply the sunscreen.  **2**. Demonstrate how to maintain motivation in response to competing demands + reinforce priority of sunscreen application. | **1**. Recognise impact of emotion on decision to adjust activities and that relationship is bi-directional.  **2**. Express confidence in ability to self-regulate own emotion to facilitate adjustment of activities  **3.** Demonstrate ability to regulate own emotion. |

Supplementary file 6. Change objectives for determinants most relevant for performance objective: *Make the decision to apply sunscreen and lip-block.*

**Determinants of photoprotection**
