# Supplementary figures and images for: Why? What? How? Using an Intervention Mapping approach to develop a personalised intervention to improve adherence to photoprotection in patients with Xeroderma Pigmentosum
Source: Health Psychol Behav Med. 2020 Oct 27;8(1):475–500. doi: 10.1080/21642850.2020.1819287 (PMC8114411; doi:10.1080/21642850.2020.1819287)

**Supplementary figure S1.** Map of social influences on students’ physical activity.

**
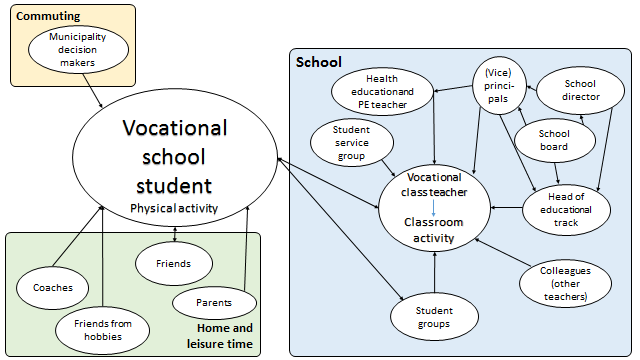
**

Supplement: Supplemental Material [file RHPB_A_1819287_SM1561.zip › suppl_data/S_Figure_S1_Map_of_social_influences_on_students_physical_activity-.docx]

**
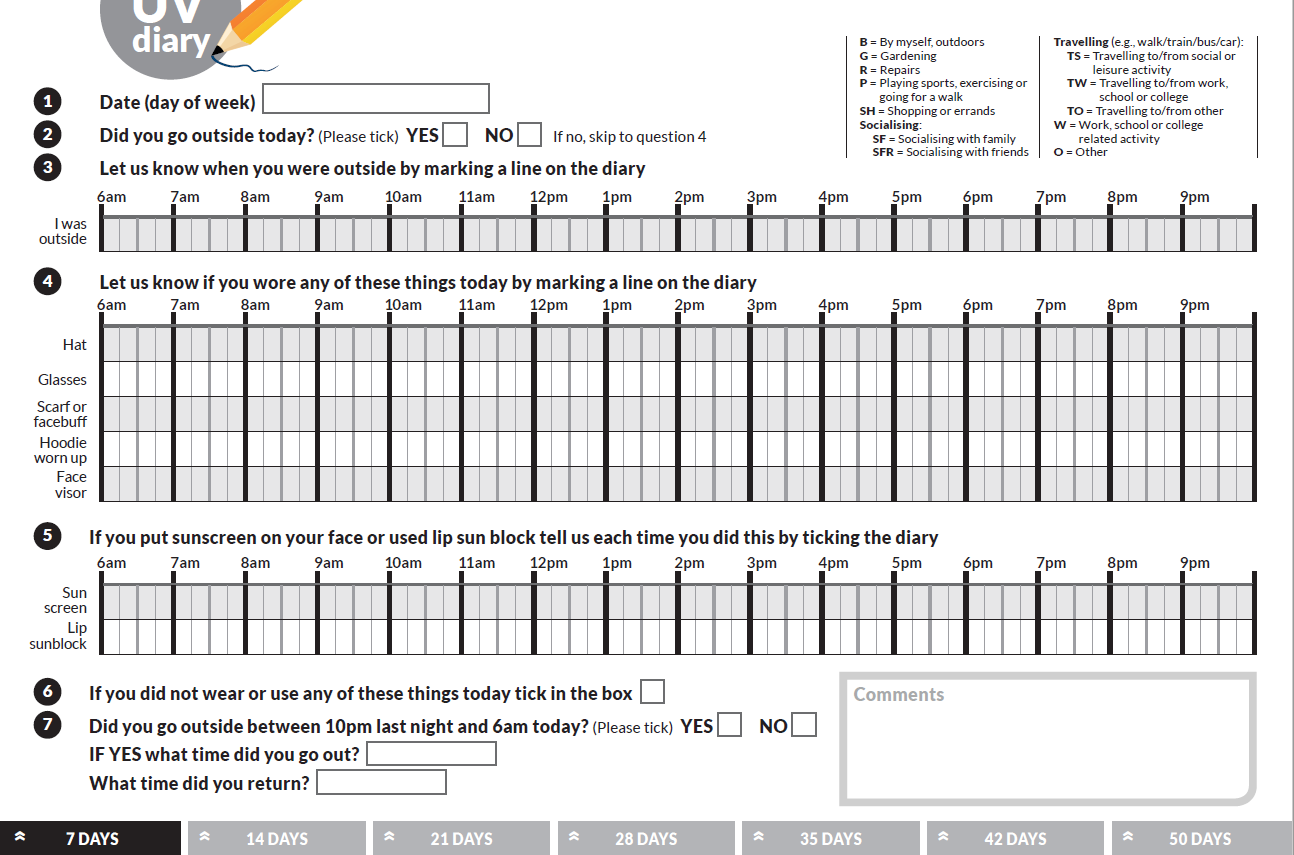
**

Supplementary file 2. UVR Protection Diary.

Supplement: Supplemental Material [file RHPB_A_1819287_SM1561.zip › suppl_data/Supplementary file 2. UVR protection diary.docx]

Supplementary file 9. UVR Dial


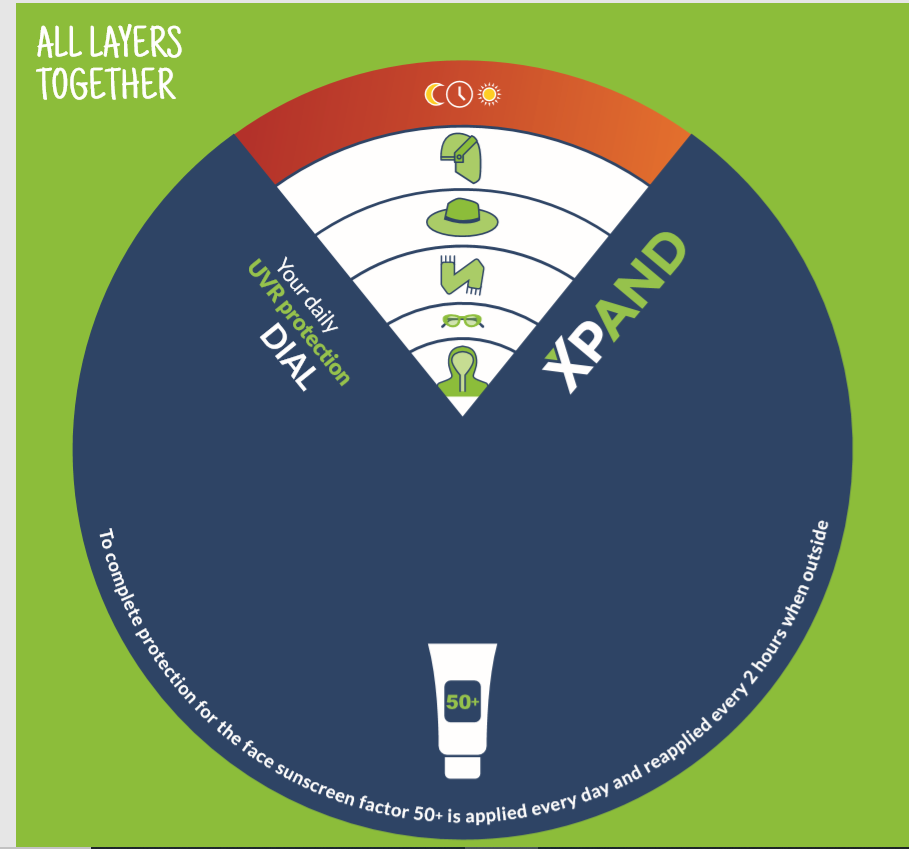

Supplement: Supplemental Material [file RHPB_A_1819287_SM1561.zip › suppl_data/Supplementary file 9. UVR dial.docx]
